# Important roles of dietary taurine, creatine, carnosine, anserine and 4-hydroxyproline in human nutrition and health

**DOI:** 10.1007/s00726-020-02823-6

**Published:** 2020-02-18

**Authors:** Guoyao Wu

**Affiliations:** Department of Animal Science and Faculty of Nutrition, Texas A&M University, College Station, TX 77843-2471 USA

**Keywords:** Amino acids, Peptides, Creatine, Metabolites, Function, Health

## Abstract

Taurine (a sulfur-containing β-amino acid), creatine (a metabolite of arginine, glycine and methionine), carnosine (a dipeptide; β-alanyl-l-histidine), and 4-hydroxyproline (an imino acid; also often referred to as an amino acid) were discovered in cattle, and the discovery of anserine (a methylated product of carnosine; β-alanyl-1-methyl-l-histidine) also originated with cattle. These five nutrients are highly abundant in beef, and have important physiological roles in anti-oxidative and anti-inflammatory reactions, as well as neurological, muscular, retinal, immunological and cardiovascular function. Of particular note, taurine, carnosine, anserine, and creatine are absent from plants, and hydroxyproline is negligible in many plant-source foods. Consumption of 30 g dry beef can fully meet daily physiological needs of the healthy 70-kg adult human for taurine and carnosine, and can also provide large amounts of creatine, anserine and 4-hydroxyproline to improve human nutrition and health, including metabolic, retinal, immunological, muscular, cartilage, neurological, and cardiovascular health. The present review provides the public with the much-needed knowledge of nutritionally and physiologically significant amino acids, dipeptides and creatine in animal-source foods (including beef). Dietary taurine, creatine, carnosine, anserine and 4-hydroxyproline are beneficial for preventing and treating obesity, cardiovascular dysfunction, and ageing-related disorders, as well as inhibiting tumorigenesis, improving skin and bone health, ameliorating neurological abnormalities, and promoting well being in infants, children and adults. Furthermore, these nutrients may promote the immunological defense of humans against infections by bacteria, fungi, parasites, and viruses (including coronavirus) through enhancing the metabolism and functions of monocytes, macrophages, and other cells of the immune system. Red meat (including beef) is a functional food for optimizing human growth, development and health.

## Introduction

The scientific conference entitled "Frontiers in Agricultural Sustainability: Studying the Protein Supply Chain to Improve Dietary Quality" hosted by New York Academy of Sciences highlighted growing controversies on meat consumption by humans in the U.S. (Wu et al. 2014). Over the past decades, there have been growing concerns that consumption of red meat (e.g., beef) increases risks for obesity, type II diabetes mellitus, cardiovascular disease, colon cancer and Alzheimer's disease in humans (e.g., Nelson et al. 2016; Willett et al. 2019). Thus, beef consumption per capita in the U.S. has steadily declined from 95 lb in 1976 to only 60 lb in 2017 (USDA 2018). This has arisen, in part, from a lack of understanding of red meat as a nutritionally important source of functional amino acids (e.g., taurine and hydroxyproline), dipeptides (e.g., carnosine and anserine), and creatine (a metabolite of amino acids) with enormous physiological significance. For example, taurine is a nutritionally essential amino acid for children (particularly preterm infants) and a conditionally essential amino acid for adults (Schaffer et al. 2009). In addition, dietary supplementation with 4-hydroxyproline improves anti-oxidative function and prevents colitis in the intestine (Ji et al. 2018). Furthermore, carnosine and anserine are potent antioxidants (Hipkiss and Gaunitz 2014), whereas creatine is both an antioxidant and a major component of energy metabolism in excitable tissues (brain and skeletal muscle) (Wyss and Kaddurah-Daouk 2000). Of note, taurine, carnosine, anserine, and creatine are particularly abundant in beef skeletal muscle but are absent from plants (Wu 2013). Also, 4-hydroxyproline is abundant in meat but negligible in many plant-source foods (Hou et al. 2019). For comparison, intramuscular concentrations of balenine (β-alanyl-l-3-methylhistidine, an antioxidant and a buffering substance) are high in whale (~ 45 mmol/kg wet weight), relatively low in swine (~ 0.7 to 1 mmol/kg wet weight or 5–8% of carnosine content), and very low in cattle, chickens and sheep (~ 0.05 to 0.1 mmol/kg wet weight; Boldyrev et al. 2013; Carnegie et al. 1982; Harris and Milne 1986). This dipeptide is barely detectable in human and rat muscles (Boldyrev et al. 2013) and, therefore, is not a focus of the present article.

Growing evidence shows that taurine, carnosine, anserine, creatine and 4-hydroxyproline play crucial roles in protecting mammalian cells from oxidative stress and injury (Abplanalp et al. 2019; Avgerinos et al. 2018; Seidel et al. 2019; Wu et al. 2019). These results indicate that red meat provides not only high-quality protein for the growth of children but also functional amino acids, dipeptides and creatine for optimal human nutrition and health. However, the public is generally not aware of these beneficial nutrients in meat, including beef (e.g., Kausar et al. 2019; Uzhova and Peñalvo 2019). To provide accurate and complete information on animal-source foods, it is imperative to review pertinent articles regarding the benefits of dietary taurine, carnosine, anserine, creatine and 4-hydroxyproline on metabolic, retinal, muscle, immunological, and cardiovascular health as well as healthy ageing in humans and animal models.

## Dietary sources of taurine, creatine, carnosine, anserine, and 4-hydroxyproline for humans

Taurine, creatine, carnosine, anserine, and 4-hydroxyproline are chemically stable and soluble in water within a physiological range of pH values. They are abundantly present in animal-source foods (such as beef). However, their reported values are highly inconsistent in the literature and may differ by 10–50 fold (Wu et al. 2016). For example, the amounts of carnosine in 100 g wet beef meat have been reported to be 14 mg (Szterk and Roszko 2014) or 1 g (Clifford 1922), and the amounts of anserine in 100 g wet beef meat to be 8.5 mg (Thornton et al. 2015) or 67 mg (Mateescu et al. 2012). Such large variations in nutrient composition may result, in part, from differences in either beef breeds or analytical techniques. To provide a much-needed database, we analyzed taurine, carnosine, anserine, creatine, and 4-hydroxyproline in cuts from three subprimals (chuck, round, and loin) of beef carcasses selected at three commercial packing plants in the United States (Table 1). These nutrients are abundant in all beef cuts (Wu et al. 2016). In contrast, taurine, carnosine, anserine, and creatine are absent from all plants [e.g., corn grains, peanuts, pistachio nuts, potatoes, soybeans, sweet potatoes, wheat flour, and white rice (Hou et al. 2019)]. Thus, vegetarians are at great risks for the deficiencies of taurine, carnosine, anserine, and creatine, particularly if they are active in physical exercise (Rogerson 2017). In addition, most of plant-source foods contained little 4-hydroxyproline and little β-alanine (a precursor of carnosine in humans) (Table 1). These data are useful for nutritionists and medical professionals to make quantitative recommendation for consumption of beef and plant-source foods by humans.

**Table 1:** The content of crude protein, amino acids, creatine, carnosine, anserine, and 4-hydroxyproline in beef and plant-source foods

Food	CP (mg/g dry weight of food)	Taurine (mg/g dry weight of food)	Carnosine (mg/g dry weight of food)	Anserine (mg/g dry weight of food)	Creatine (mg/g dry weight of food)	OH-Pro (mg/g dry weight of food)	β-Alanine (mg/g dry weight of food)	Glycine (mg/g dry weight of food)
Beef cut—chuck	680	2.34	15.2	2.79	9.60	1.73	0.453	31.0
Beef cut—round	721	2.78	21.4	3.25	10.2	1.74	0.615	33.3
Beef cut—loin	734	2.92	24.2	3.66	10.5	1.77	0.712	33.7
Corn	101	0.00	0.00	0.00	0.00	0.04	0.010	4.43
Potato	98.4	0.00	0.00	0.00	0.00	0.08	0.046	2.74
Soybean	446	0.00	0.00	0.00	0.00	0.78	0.069	24.0
Wheat flour	134	0.00	0.00	0.00	0.00	0.43	0.0040	6.31
White rice	82.5	0.00	0.00	0.00	0.00	0.04	0.0024	3.95

Adapted from Wu et al. (2016), Wu et al. (2020), and Hou et al. (2019)

*CP* crude protein (6.25  ×  N%), *OH-Pro* 4-hydroxyproline

## Biochemistry, physiology, and nutrition of taurine, creatine, carnosine, anserine, and 4-hydroxyproline in humans

### Taurine

#### Tissue distribution of taurine

Taurine (2-aminoethanesulfonic acid, a slightly acidic substance) is a sulfur-containing β-amino acid. It was originally isolated from the bile salts (taurocholate, also known as cholyltaurine) of the ox by Austrian scientists F. Tiedermann and L. Gmelin in 1827. Taurine is widely distributed in the animal kingdom and to be present in relatively high concentrations in all mammalian and avian tissues, such as blood, intestine, liver, skeletal muscle, heart, brain, kidneys, and retina. A 70-kg person has ~ 70 g taurine (HuxTable 1992). Concentrations of taurine in mammalian and avian cells range from 5 to 60 mM, depending on species and cell type (Wright et al. 1986). Human skeletal muscle, heart, retina, and placenta contain 15–20, 28–40, 20–35, and 20–35 mM taurine, respectively. Because of its large mass [e.g., accounting for 45% of body weight (BW) in healthy, non-obese adults], skeletal muscle is the major site (about 70%) of taurine storage in the 70-kg adult. Taurine is abundant in the milk of mammals (e.g., 0.4 mM in human, 0.8 mM in mouse, and 2.8 mM in cats) as a physiologically essential nutrient for infants and children (Sturman 1993).

#### Absorption of taurine by the small intestine and transport in blood

**Fig. 1:**
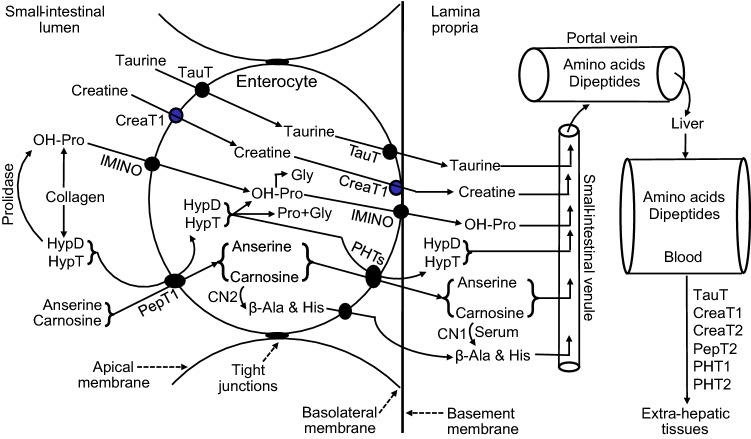
Absorption of taurine, creatine, carnosine, anserine, and 4-hydroxyproline by the human small intestine and the transport of the nutrients in blood. Dietary collagen is hydrolyzed by proteases, peptidases and prolidase to free amino acids as well as 4-hydroxyproline and its peptides. Dietary taurine, creatine, carnosine, anserine, and 4-hydroxyproline are taken up by the enterocyte across its apical membrane via specific transports. Inside the cell, taurine, creatinine and anserine are not degraded, some of the 4-hydroxyproline-containing peptides are hydrolyzed to 4-hydroxyproline and its peptides, some 4-hydroxyproline is oxidized to glycine, and carnosine undergoes limited catabolism. Taurine, creatine, carnosine, anserine, and 4-hydroxyproline, as well as the products of carnosine hydrolysis (β-alanine and histidine) exit the enterocyte across its basolateral membrane into the lamina propria of the intestinal mucosa via specific transporters (Wu 2013). The absorbed nutrients are transported in blood in the free forms for uptake by extra-intestinal tissues via specific transporters. *β-Ala* β-alanine, *CAT* cationic amino acid transporter, *CN1* carnosinase-1 (serum carnosinase), *CN2* carnosinase-2 (tissue carnosinase), *CreaT1* creatine transporter-1, *CreaT2* creatine transporter-2, *GAT* γ-aminobutyrate transporter, *HypD* 4-hydroxyproline-containing dipeptides, *HypT* 4-hydroxyproline-containing tripeptides, *OH-Pro* 4-hydroxyproline, *PAT1* proton-(H^+^-coupled) and pH-dependent but Na^+^- and Cl^−^-independent transporter for taurine (low-affinity, high-capacity transporter), *PepT1* peptide transporter-1, *PepT2* peptide transporter 2, *PHT1/2* peptide/histidine transporters 1 and 2, *TauT* taurine transporters. Note that the distribution of PHT1/2 in tissues is species-specific in that human skeletal muscle expresses PHT1 but no PHT2, whereas mouse skeletal muscle expresses both PHT1 and PHT2

Dietary taurine is taken up by the enterocyte across its apical membrane via Na^+^- and Cl^−^-dependent transporters, TauT, GAT2 and GAT3 (Lambert and Hansen 2011; Zhou et al. 2012), as well as PAT1 (a H^+^-coupled, pH-dependent but Na^+^- and Cl^−^-independent transporter), with TauT being the major transporter under physiological conditions (Anderson et al. 2009). Taurine exits the enterocyte across its basolateral membrane into the lamina propria of the intestinal mucosa via specific transporters (Fig. 1). Absorbed taurine is not degraded by the intestinal mucosa and, therefore, enters the portal circulation. Taurine is transported in blood as a free amino acid for uptake by extra-intestinal tissues (Wu 2013). Increasing dietary intake of taurine enhances its concentrations in plasma and tissues, such as skeletal muscle, brain and heart (Schaffer et al. 2009; Sirdah 2015).

#### Synthesis of taurine in humans

Taurine is synthesized from cysteine, a product of methionine catabolism, in the liver of many mammals (Wu 2013). In humans, the rate of taurine synthesis from methionine and cysteine is exceedingly low in comparison with rats (Sturman 1993), because the activity of hepatic cysteinesulfinate decarboxylase (a key enzyme in taurine synthesis) is about three orders of magnitude lower in the young and adult men than that in rats (Sturman and Hayes 1980). Compared with livestock (e.g., cattle, pigs, and sheep) and poultry (e.g., chickens and ducks), humans also have a very low ability to synthesize taurine at any stage of life. Depending on the dietary intake of protein, nutritional status, and hepatic enzyme activity, a healthy adult may synthesize 50–125 mg taurine per day (Jacobsen and Smith 1968). Under stress or diseased conditions (e.g., heat stress, infection, obesity, diabetes, and cancer), taurine synthesis in the body may be impaired due to the suboptimal function of liver and the reduced availability of the amino acid precursors. Infants and children do not sufficiently synthesize taurine despite adequate provision of its precursors in diets (Geggel et al. 1985). In addition, individuals who consume only plant-source foods but no animal products are at increased risk for taurine deficiency, because the precursors of taurine (methionine and cysteine) are present at low concentrations in most proteins of plant origin (e.g., corn, potato, rice, wheat, and vegetables) (Hou et al. 2019).

#### Metabolism of taurine in humans

In animal cells, taurine undergoes limited catabolism by taurine:pyruvate transaminase or taurine:α-ketoglutarate transaminase, which catalyzes the transamination of taurine with pyruvate or α-ketoglutarate to form 2-sulfoacetaldehyde (also known as 2-oxoethanesulfonate, 2-hydroxyethanesulfonate, or isethionate) and l-alanine or l-glutamate (Read and Welty 1962). These reactions also occur in aerobic and anaerobic bacteria (Cook and Denger 2006). In addition, taurine dehydrogenase converts taurine into ammonia plus 2-sulfoacetaldehyde (an oxidation reaction) in aerobic bacteria in the presence of cytochrome C as the physiological electron acceptor (Brüggemann et al. 2004). Furthermore, taurine is oxygenated by α-ketoglutarate-dependent taurine dioxygenase to generate sulfite and 2-aminoacetaldehyde in microorganisms (including *E. coli* Eichhorn et al. 1997). The initial fate of the degradation of the taurine's sulfur atom is sulfite in strictly anaerobic, facultatively anaerobic, or strictly aerobic bacteria (Brüggemann et al. 2004). Thus, taurine catabolism is initiated by transamination, oxidation, or oxygenation in a species and cell-specific manner.

**Fig. 2:**
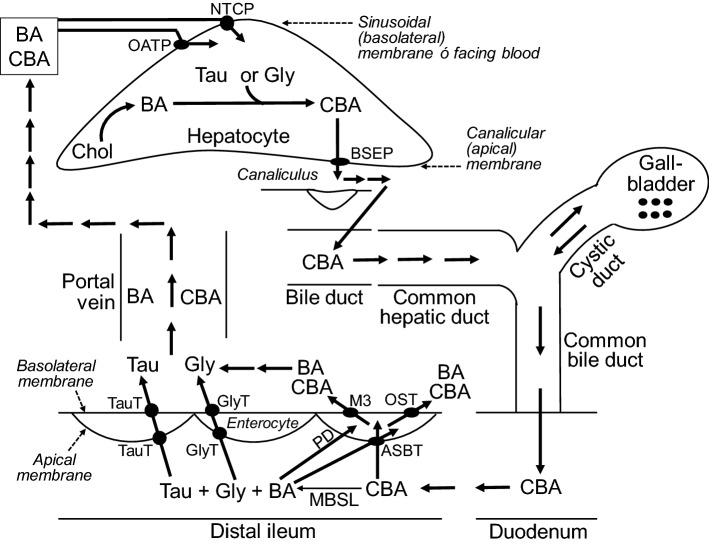
The transport of bile salts from the liver to the duodenum and the return of bile salt from the distal ileum to the liver via the enteral-hepatic circulation in humans. Conjugated bile acids are exported by the ATP-dependent bile salt export pump out of the hepatocyte through its canalicular (apical) membrane into the canaliculus. The bile salts subsequently enter bile ducts, the common hepatic duct, and the gallbladder. During digestion, the bile salts are secreted from the gallbladder to the common bile duct and then the duodenum. In the distal ileum, a fraction of bile salts is hydrolyzed by microbial bile salt hydrolases to form bile acids and taurine or glycine. Taurine, glycine and bile salts are efficiently taken up by the enterocytes of the distal ileum via specific transporters. The substances are transported in the blood for uptake by the hepatocyte via its sinusoidal basolateral membrane. During each enteral-hepatic cycle, about 95% of the liver-derived bile salts are reabsorbed to the liver. *ASBT* apical sodium-dependent bile salt/acid transporter (in ileal enterocytes), *BA* bile acids (unconjugated), *BSEP* bile salt export pump, *CBA* conjugated bile acids, *Gly* glycine, *GlyT* glycine transporters, *M3* multidrug resistance protein-3, *MBSL* microbial bile salt hydrolases, *NTCP* Na^+^-taurocholate cotransporting polypeptide, *OATP* organic anion transporting polypeptide family, *OSTα/β* organic solute transporter subunit α/β, *PD* passive diffusion, *Tau* taurine, *TauT* taurine transporters

In humans, taurine is covalently conjugated with bile acids in the liver to form bile salts, which are then exported by the ATP-dependent bile salt export pump out of the hepatocyte and stored in the gallbladder (Hofmann 1999). During feeding, bile salts are secreted from the gallbladder via the common bile duct into the duodenum, where they facilitate the digestion and absorption of dietary lipids. The bile salts are not absorbed by the proximal small intestine (duodenum, jejunum and proximal ileum) due to the lack of their apical transporters, and are resistant to deamidation by pancreatic and mucosal enzymes (including peptidases). Instead, after the absorption of dietary lipids, bile salts enter the distal ileum, where a small fraction of them are hydrolyzed (and thus deconjugated) by microbial bile salt hydrolases to form bile acids and taurine (Foley et al. 2019). Taurine, the remaining large proportion of bile salts, and bile acids are efficiently absorbed into the enterocyte of the distal ileum by specific transporters (Figs. 1, 2). About 95% of the liver-derived bile salts and bile acids are absorbed primarily by the distal ileum into the portal circulation, and then taken up by hepatocytes of the liver (Fig. 2). This is known as the enterohepatic circulation for the ileal reabsorption of bile salts and bile acids. Only ~ 5% of the liver-derived bile salts and bile acids enter the large intestine during each enterohepatic circulation. Thus, it generally takes a prolonged period of time (e.g., months) to induce a taurine deficiency in humans and animals.

#### Physiological functions of taurine

It is now recognized that taurine plays major roles in human physiology and nutrition, including serving as: (1) a nutrient to conjugate bile acids to form bile salts in the liver that facilitate intestinal absorption of dietary lipids (including lipid-soluble vitamins) and eliminate cholesterol in bile via the fecal route; (2) a major antioxidant, anti-inflammatory, and anti-apoptotic factor in the body; (3) a physiological stabilizer of cell membranes; (4) a regulator of modulation of Ca^2+^ signaling, fluid homeostasis in cells, and retinal photoreceptor activity; (5) a contributor to osmoregulation; (6) a key component of nerve and muscle conduction networks; (7) a stimulator of neurological development; and (8) an inhibitory neurotransmitter in the central nervous system (CNS) (Schaffer and Kim 2018). Thus, taurine exerts beneficial effects on cardiovascular (including protection against ischemia–reperfusion injury, maintaining cell membrane structure, and reducing blood pressure), digestive, endocrine, immune, muscular, neurological, reproductive, and visual systems (Ito et al. 2014; Shimada et al. 2015; Seidel et al. 2019). For example, taurine protects cells and tissues (brain) from the toxicity of reactive oxygen species (ROS), excess metals [e.g., nickel (Xu et al. 2015) and manganese (Ahmadi et al. 2018)], and ammonia (Jamshidzadeh et al. 2017a) by maintaining the integrity of plasma and organelle (especially mitochondrion) membranes of the cell. Conversely, a deficiency of dietary taurine results in retinal degeneration in cats (Hayes et al. 1975) and children (Geggel et al. 1985) that can be corrected with taurine supplementation. Furthermore, through the production of N-chlorotaurine by activated human granulocytes and monocytes, taurine contributes to the killing of pathogenic bacteria, fungi, parasites, and viruses (Gottardi and Nagl 2010).

#### Health benefits of taurine supplementation

Since 1975, taurine has been used for 45 years as a dietary supplement to improve health in humans and animal models of metabolic syndrome (El Idrissi 2019; Hayes et al. 1975; Ra et al. 2019). For example, taurine supplementation can prevent diabetic rats from developing cardiomyopathy by inhibiting the expression of the angiotensin II type-2 receptor (Li et al. 2005), modulating mitochondrial oxidative metabolism (Militante et al. 2000) and electron transport activity (Jong et al. 2012), and reducing oxidative stress (Schaffer et al. 2009). In addition, oral administration of taurine can ameliorate structural abnormalities of the pancreas (Santos-Silva et al. 2015), while improving insulin production by pancreatic β-cells (Nakatsuru et al. 2018) and whole-body insulin sensitivity in subjects with hyperglycemia (Sarkar et al. 2017). This role of taurine in diabetic patients are highly significant, because they have a 25% lower concentration of taurine in plasma than normal subjects (Sak et al. 2019) and that the number of type-2 diabetic patients is increasing globally (Willet et al. 2019). Taurine supplementation may also be needed for cancer patients maintained on total parenteral nutrition, because the concentration of taurine in their plasma is 50% lower than those in healthy subjects (Gray et al. 1994). Of particular note, ingestion of 0.4–6 g taurine per day for various days improved (1) metabolic profiles in blood (including reductions in total cholesterol and low-density-lipoprotein cholesterol), and (2) cardiovascular functions in healthy subjects, as well as in patients with overweight, diabetes, hypertension, or congestive heart failure (Militante and Lombardini 2002; Xu et al. 2008). In addition, oral administration of 2 g taurine/day for 4 weeks resulted in clinically significant reductions in the frequency, duration, and intensity of muscle cramps in patients with chronic liver disease (Vidot et al. 2018). Furthermore, long-term oral administration of taurine (9 or 12 g per day) for 52 weeks can effectively reduce the recurrence of stroke-like episodes in mitochondrial myopathy, encephalopathy, lactic acidosis and stroke-like episodes (MELAS), a rare genetic disorder caused by point mutations in the mitochondrial DNA (Ohsawa et al. 2019). These beneficial effects of oral taurine are summarized in Table 2.

**Table 2:** Beneficial effects of dietary taurine, creatine, carnosine, anserine and 4-hydroxyproline on human health

Dosage	Subjects	Benefits	System	References
Taurine				
1.5 to 2.25 g/day for 12 weeks	Children maintained on long-term PNWT	Prevention of electroretinographic abnormality	Retinal health	Geggel et al. (1985)
0.4 or 1.6 g/day for 2 weeks	Healthy adults	Decreases in platelet aggregation and thromboxane release from platelets	Cardiovascular health	Xu et al. (2008)
1.5 g/day for 90 days	Patients with IDDM	Increase in platelet taurine and a decrease in platelet aggregation	Cardiovascular health	Xu et al. (2008)
3 g/day for 7 weeks	Overweight or obese adults	Decreases in body weight, plasma TAGs, and atherogenic index	Cardiovascular and metabolic health	Xu et al. (2008)
3 g/day for 60 days	Adults with mild or borderline HPT	Decrease in blood pressure	Cardiovascular health	Xu et al. (2008)
3 g/day for 30 to 45 days	Patients with CHF	Decrease in left ventricular end-diastolic volume and increase in serum creatinine	Cardiovascular and metabolic health	Xu et al. (2008)
3 g/day for 6 weeks	Patients with CHF	Improvement in cardiac function	Cardiovascular health	Xu et al. (2008)
6 g/day for 3 weeks	Healthy adults fed a high-fat diet (40% energy from fats)	Decreases in total cholesterol, LDL, and LDL-cholesterol coentrations in serum	Cardiovascular and metabolic health	Xu et al. (2008)
6 g/day for 1 week	Patients with hypertension	Decrease in blood pressure	Cardiovascular health	Militante and Lombardini (2002)
2 g/day for 4 weeks	Patients with chronic liver disease	Amelioration of muscle cramps	Skeletal muscle and metabolic health	Vidot et al. (2018)
9–12 g/day for 52 weeks	Patients with MELAS	Reduce the recurrence of stroke-like episodes	Cardiovascular and metabolic health	Ohsawa et al. (2019)
1–6 g/day for up to 2 weeks	Healthy adults	Improve endurance exercise performance	Skeletal muscle and metabolic health	Waldron et al. (2018)
Creatine (Cr) in the form of Cr monohydrate (CrM) as Cr phosphate (CrP)				
20 g CrM/day for 6 days; 2 g CrM/day for 22 days	Healthy adult men	Improve muscular strength and reduce intensive exercise-associated muscle damage	Skeletal muscle health	Wang et al. (2018)
4 g CrM/day for 6 weeks	Healthy adult men and women	Enhance anaerobic power and strength	Skeletal muscle health	Hummer et al. (2019)
4 g CrM/day for 6 weeks	Healthy adult men	Enhance sprint cycling performance	Skeletal muscle health	Crisafulli et al. (2018)
10–20 g CrM or CrP/day for 6 weeks^a^	Healthy adult men	Enhance muscular strength and lean-tissue mass in the body	Skeletal muscle and metabolic health	Peeters et al. (1999)
3 g CrM/day for weeks or months	Healthy adult men and women	Improve anti-oxidative capacity, exercise performance and recovery	Skeletal muscle and metabolic health	Kreider et al. (2017)
3 g CrM/day for weeks or months	Patients with neurodegenerative diseases	Improve neurological and muscular function	Neurological and muscle health	Kreider et al. (2017)
Carnosine				
116 mg/day for 8 weeks	Patients with gastric ulcers	Enhance gastric healing	Gastric health	Sakae and Yanagisawa (2014)
1.5 g/day for 30 days	Patients with Parkinson’s disease	Improve neurological function	Neurological health	Boldyrev et al. (2008)
2 g/day for 3 months	Patients with schizophrenia	Improve neurological function	Neurological health	Chengappa et al. (2012)
0.8 g/day for 8 weeks	Patients with austic spectrum disorder	Improve behavior as well as social and communication skills	Neurological health	Chez et al. (2002)
1 g/day for 12 weeks	Patients with type-2 diabetes	Improve metabolic profiles; decrease protein glycosylation and body fat	Cardiovascular and metabolic health	Houjeghani et al. (2018)
2 g/day for 12 weeks	Overweight or obese subjects	Improve metabolic profiles; increase lean-tissue mass in the body	Cardiovascular and metabolic health	de Courten et al. (2016)
0.5 to 2 g/day for up to 6 months	Patients with heart failure	Enhance cardiac output and improve the quality of life	Cardiovascular and metabolic health	Cicero and Colletti (2017)
Anserine				
10 or 100 mg/45 kg body weight	Adults undergoing OGTT	Reduce glucose concentration in blood	Metabolic health	Kubomura et al. (2010)
Anserine and carnosine mix^b^	Elderly subjects	Maintain adequate blood flow to the brain, preserve verbal episodic memory, and improve resting-state network connectivity	Neurological health	Ding et al. (2018); Rokicki et al. (2015)
Anserine and carnosine mix^b^	Elderly subjects	Attenuate cognitive impairment	Neurological health	Masuoka et al. (2019)
Anserine and carnosine mix^b^	Elderly subjects	Inhibit the production of inflammatory cytokines	Metabolic health and immunity	Katakura et al. (2017)
Anserine and carnosine mix^b^	Elderly subjects	Enhance muscular strength and exercise performance	Skeletal muscle health	Hirohiko et al. (2006)
0.01 to 0.6 g/day for weeks or months	Adults	Ameliorate stress; enhance physical strength; improve metabolic profiles, immunity, neurological function, and wound healing; promotes lactation	Endocrine, metabolic, immune, skeletal muscle, and neurological health	Li et al. (2012); Szcześniak et al. (2014)
4-Hydroxyproline in the form of collagen hydrolysate (CH)				
2.5 or 5 g CH/day for 8 weeks	Adult women	Improve skin elasticity	Skin health	Proksch et al. (2014)
5 g CH/day for 6 weeks	Adult women	Enhance moisture content in the epidermis	Skin health	Matsumoto et al. (2006)
10 g CH/day for 8 weeks	Adult women	Improve collagen density in the dermis and the structure of collagen network	Skin health	Asserin et al. (2015)
5 g CH/day for 8 weeks	Adult women	Improve facial skin conditions	Skin health	Inoue et al. (2016)
5 g CH/day for 1 year	Postmenopausal women	Improve mineral density in bones	Bone health	König et al. (2018)
10 g CH/day for 24 weeks	Postmenopausal women	Mitigate osteoporosis	Bone health	Adam et al. (1996)
10 g CH/day for 60 days	Subjects with knee osteoarthritis	Ameliorate joint pain	Bone health	Deal and Moskowitz (1999)

There were no side effects for the ingestion of taurine, creatine, carnosine, anserine and hydroxyproline at the indicated dosages on all the studies

*CHF* congestive heart failure, *HPT* hypertension, *IDDM* insulin-dependent diabetes mellitus, *LDL* low-density lipoproteins, *MELAS* mitochondrial myopathy, encephalopathy, lactic acidosis and stroke-like episodes, *OGTT* oral glucose tolerance test, *PNWY* parenteral nutrition (supply of nutrients through intravenous infusion) without taurine, *TAGs* triacylglycerols, *CH* collagen hydrolysate, *Cr* creatine, *CrM* creatine monohydrate, *CrP* creatine phosphate

^a^20 g CrM or CrP/day for 3 days, followed by 10 g CrM or CrP/day for 39 days

^b^1 g of anserine and carnosine mix per day, 3:1 (g/g), for 3 months

Waldron et al. (2018) conducted a meta-analysis to evaluate the effects of oral ingestion of taurine at various doses on endurance performance in adult humans who consumed 1–6 g taurine/day in single doses for up to 2 weeks. The authors found that the ingestion of  taurine improved overall endurance performance in the subjects. This conclusion is consistent with the recent findings that taurine is a potential ergogenic aid for preventing muscle damage, attenuating muscle protein catabolism, decreasing oxidative stress, and improving performance in subjects with endurance exercise (De Carvalho et al. 2017; Page et al. 2019; Paulucio et al. 2017; Waldron et al. 2019).

### Creatine

#### Tissue distribution of creatine

Creatine (*N*-[aminoiminomethyl]-*N*-methyl glycine), “*kreas*” in Greek meaning meat, was discovered by the French chemist Michel E. Chevreul in 1832 as a component of skeletal muscle in cattle. This neutral, water-soluble substance is abundant in skeletal muscle, heart, brain, and pancreas. A 70-kg adult has about 120 g of total creatine (creatine phosphate plus free creatine), with about 95% of it being in skeletal muscle (Casey and Greenhaff 2000). Total creatine is about 45% more abundant in white (fast-twitch fibers, type II) muscle than in red (slow-twitch fibers, type I) muscle (Murphy et al. 2001). In skeletal muscle and brain, creatine stores energy (primarily ATP) as creatine phosphate through the action of creatine kinase. In a resting state, about two-third and one-third of total creatine exist as creatine phosphate and creatine, respectively (McGilvery and Murray 1974). The irreversible loss of creatine as creatinine is 1.7% of the whole-body creatine (Brosnan and Brosnan 2007). This means that each day, 98.3% of creatine is continuously recycled to store ATP energy via creatine kinase. In human skeletal muscle, the concentration of creatine phosphate is about three to four times that of ATP (Gray et al. 2011; McGilvery and Murray 1974). The energy of the γ-phosphate bond (51.6 kJ/mol or 12.3 kcal/mol) in one mole of ATP is transferred to one mole of creatine for storage in vivo (Wu 2018).

#### Absorption of creatine by the small intestine and transport in blood

In the small intestine of mammals including humans, the apical membrane of its enterocytes absorb dietary (luminal) creatine via Na^+^/Cl^−^-coupled (i.e., sodium- and chloride-dependent) creatine transporter-1 (CreaT1) (Santacruz and Jacobs 2016). All the dietary-free creatine (100%) is absorbed by the small intestine of humans into the portal circulation (Deldicque et al. 2008). Ingested creatine phosphate, which is also abundant in meat, is hydrolyzed extracellularly by alkaline phosphatases (synthesized and released by enterocytes) into creatine and phosphate before intestinal absorption (Fernley 1971). Absorbed creatine undergoes limited phosphorylation (only 1% of ingested creatine) in the intestinal mucosa, and nearly 99% of orally ingested creatine enters the portal circulation (Jäger et al. 2011). Creatine is transported in blood as a free substance, and is rapidly taken up by extra-intestinal tissues and cells (e.g., liver, skeletal muscle, brain, kidneys, testes, and other tissues and cell types) via CreaT1, and by testes and brain also via CreaT2 (Bala et al. 2013). CreaT2 protein shares 97% homology with CreaT1. Immunohistochemical analysis has shown that CreaT1 is mainly associated with the sarcolemmal membrane in all types of skeletal muscle (Christie 2007; Murphy et al. 2001). CreaT1 is widespread in animal tissues, whereas CreaT2 is primarily present within the testes (Snow and Murphy 2001). CreaT1 is essential for normal brain and muscular function as mutations in the gene (SLC6A8) result in X-linked mental retardation, severe speech and language delay, epilepsy, autistic behavior, and muscular abnormalities (e.g., hypotonia) (Bala et al. 2013; Salomons et al. 2001). Increasing dietary intake of creatine enhances its concentrations in plasma and tissues, such as skeletal muscle, brain and heart (Derave et al. 2004; Wyss and Kaddurah-Daouk 2000). The absorption of creatine by the small intestine and its transport in the blood are illustrated in Fig. 1.

#### Synthesis of creatine in humans

Creatine is a metabolite of three amino acids (arginine, glycine and methionine) via the cooperation of multiple organs, primarily including the liver, pancreas and kidneys (Wu 2013). Beef is an abundant source of arginine, glycine and methionine. In contrast, all plant-source foods contain low levels of glycine and methionine, and most of plant-source foods (except for soybean, peanuts and other nuts) also have low levels of arginine (Hou et al. 2019). The pathway of creatine synthesis is initiated by arginine:glycine amidinotransferase, which transfers the guanidino group from arginine to glycine to form guanidinoacetate and ornithine. Arginine:glycine amidinotransferase is expressed primarily in the renal tubules, pancreas, and to a much lesser extent in the liver and other organs. Thus, the kidneys are the major site of guanidinoacetate formation in the body. The guanidinoacetate released by the kidneys is methylated by guanidinoacetate *N*-methyltransferase, which is located predominantly in the liver, pancreas, and, to a much lesser extent, in the kidneys to produce creatine (Wu and Morris 1998).

Creatine synthesis is regulated primarily through: (1) changes in expression of renal arginine: glycine amidinotransferase in both rats and humans; and (2) the availability of substrates. Dietary intake of creatine and circulating levels of growth hormone are major factors affecting de novo synthesis of creatine (Wu and Morris 1998). Activities and mRNA levels for arginine:glycine amidinotransferase in rat kidney are greatly reduced by hypophysectomy or by feeding a diet containing creatine. Neither creatine supplementation nor growth hormone influences the hepatic activity of guanidinoacetate *N*-methyltransferase in animals. Thus, dietary supplementation with creatine helps to spare arginine, glycine and methionine for utilization via other vital metabolic pathways, such as the syntheses of protein, nitric oxide, and glutathione (Wu 2013). This has important nutritional and physiological significance.

A 70-kg healthy adult synthesizes 1.7 g creatine per day from 2.3 g arginine, 1.0 g glycine, and 2.0 g methionine (Wu and Morris 1998), which represent 46%, 36% and 87%, respectively, of their daily dietary intakes. This amount of creatine is necessary to replace its daily irreversible loss (1.7 g/day) as creatinine from the subject through the excretion of urine. A greater loss of creatine from the body occurs in response to enhanced muscular activity (Kreider et al. 2017; Rogerson 2017), and this amount of creatine should be replenished through enhanced endogenous synthesis and dietary supplementation. For example, cycle ergometer exercise (approximately 45% of maximum O_2_ consumption for 90 min) increases the loss of creatine (as indicated by urinary creatinine excretion) by 52% above pre- and post-exercise values (Calles-Escandon et al. 1984).

There are new lines of evidence that endogenous synthesis of creatine is not sufficient for humans under many physiological (e.g., exercise, pregnancy and lactation) and pathological (e.g., tissue injury, ischemia, and diabetes) conditions. First, dietary creatine supplementation aids in increasing the power, strength, and mass of the skeletal muscle in athletes and bodybuilders, especially those who consume little meat (Smith et al. 2014). Second, creatine supplementation is beneficial for ameliorating sarcopenia in elderly subjects that is characterized by reductions in the mass and function of skeletal muscle (Candow et al. 2014). Third, patients with neurological and muscular disorders can respond well to creatine supplementation with improvements in health, as summarized previously. Thus, adequate provision of dietary creatine may be necessary for maintaining homeostasis and optimal health in humans (Brosnan and Brosnan 2007), particularly for vegan athletes who generally have low intake of creatine and its precursors (arginine, methionine and glycine) (Rogerson 2017).

#### Metabolism of creatine in humans

Creatine undergoes limited catabolism in mammalian cells (Wu 2013). However, creatine is reversibly converted by creatine kinase into creatine phosphate and is irreversibly cyclized into creatinine through spontaneous loss of one molecule of water, as indicated previously. Like creatinine, creatine phosphate is also spontaneously converted into creatinine. The breakdown of creatine phosphate results in the losses of phosphate and one molecule of water. Creatine kinase exists in the cytosolic and mitochondrial isoforms. This enzyme is not only most abundant in skeletal muscle and brain, but is also present in many tissues and cell types (Keller and Gordon 1991; Trask and Billadello 1990). When healthy adults consume 4.4 g creatine monohydrate once, plasma creatine peaks [762 µM; an 18-fold increase over the 0-min baseline value of about 40 µM (Jäger et al. 2007)] in 60 min (Kreider et al. 2017) and its half-life is 30 min (Jäger et al. 2007). Thus, creatine is rapidly cleared from plasma, and skeletal muscle is the major sink of creatine. Creatinine is excreted in urine. The amount of urinary creatinine is proportional to (and, therefore, an indicator of) skeletal muscle mass in healthy subjects.

#### Physiological functions of creatine

Creatine is essential for energy metabolism in the brain and skeletal muscle (Wyss and Kaddurah-Daouk 2000). In these two highly excitable tissues that can undergo rapid changes in their membrane potentials for the transport of electrical signals, creatine decreases intracellular calcium concentrations (e.g., by stimulating sarcoplasmic reticulum Ca^2+^-ATPase in skeletal muscle) and extracellular glutamate concentrations (e.g., through uptake by synaptic vesicles in the brain), and prevents the opening of the mitochondrial permeability transition pore (Bender et al. 2005; Fortalezas et al. 2018). Creatine also plays an important role in anti-oxidative and anti-apoptotic reactions, scavenging free radicals, and protection against excitotoxicity in tissues (Lawler et al. 2002). For example, creatine can prevent mitochondrial disorders that are commonly associated with decreased ATP production and increased ROS production (Rodriguez et al. 2007; Wyss and Kaddurah-Daouk 2000).

#### Health benefits of creatine supplementation

By delaying the depletion of ATP during oxygen deprivation and reducing the availability of ROS (Balestrino et al. 1999), pretreatment with creatine can reduce the cardiac and neurological damages induced by ischemia or anoxia and that such treatment can also be useful even after the onset of stroke or myocardial infarction (Balestrino et al. 2002; Lensman et al. 2006; Osbakken et al. 1992; Perasso et al. 2013; Prass et al. 2007; Scheer et al. 2016; Shen and Goldberg 2012; Whittingham and Lipton 1981; Wilken et al. 1998; Zapara et al. 2004). Of note, creatine has been safely and beneficially administered to patients affected by age-related neurological diseases, including Parkinson's disease, Huntington's disease, amyotrophic lateral sclerosis, long-term memory deficits, Alzheimer's disease, and stroke (Adhihetty and Beal 2008; Smith et al. 2014), as well as patients with pathophysiological conditions, such as gyrate atrophy, post-stroke depression, congestive heart failure, chronic musculoskeletal pain disorders, atherosclerotic diseases, and cisplatin-induced renal damage (Genc et al. 2014; Hummer et al. 2019; Persky and Brazeau 2001; Wyss and Schulze 2002). Through increasing the availability of arginine for the generation of nitric oxide (a killer of pathogenic bacteria, fungi, parasites, and viruses), dietary supplementation with creatine plays an important role in protecting humans from infectious diseases (Ren et al. 2018).

Athletes and muscle builders are major users of supplemental creatine. This is primarily because (1) both energy metabolism and production of oxidants are enhanced during exercise, and (2) creatine participates in ATP turnover in skeletal muscle and is a potent antioxidant. Due to the very low rate of creatine loss (1.7%) in the whole-body pool (creatine plus creatine phosphate), dietary supplementation with a low dose of creatine (e.g., 3 g of creatine monohydrate per day for 28 days in 76-kg men) results in a steady accumulation of creatine in skeletal muscle, and the saturation of creatine in the muscle (142 mmol/kg dry matter) occurs by day 28 (Hickner et al. 2010; Hultman et al. 1996). For comparison, the content of creatine in the skeletal muscle of adult humans without creatine supplementation is 122 mmol/kg dry matter of muscle (Hultman et al. 1996). Thus, based on the current knowledge of creatine metabolism in humans, continuous supplementation with creatine is not necessary to achieve the saturation of creatine in skeletal muscle. In the case of discontinuous supplementation of creatine, such as disruption for short intervals (e.g., 1 or 2 days per week), a longer period of supplementation (e.g., by 5–10 days) should still be sufficient to achieve the saturation of creatine in skeletal muscle. Thus, consumption of creatine monohydrate (3 g/day) over a prolonged period of time can beneficially increase the concentration of creatine in skeletal muscle to the saturation level even if the supplement is not taken every single day. When an adult consumes daily 30 g of dry beef that provides 303 mg creatine (Wu et al. 2016) as its sole dietary source, it will take about 240 days to saturate creatine in skeletal muscle.

Because of increased losses of creatine during active muscular work as noted previously, oral administration of creatine is necessary for maintaining its homeostasis in exercising subjects and this nutritional strategy is beneficial for improving their performance. For example, dietary supplementation with a low dose of creatine (as 3 g of creatine monohydrate per day) to adults can enhance the capacity of skeletal muscle to store energy by 16% [(142 – 122)/122 = 16%] (Hultman et al. 1996). The rate of utilization of creatine phosphate by the skeletal muscle of adult humans during intensive exercise is 23 mmol/L of muscle water/min (Baker et al. 2010). This is equivalent to 483 mmol/whole-body skeletal muscle/min (23 × 70 × 40% × 0.75 = 483) for a 70-kg adult with 21 L of muscle water. Through creatine recycling via creatine kinase, ingestion of 3 g of creatine monohydrate can help store 50 kcal energy in the skeletal muscle of a 70-kg person for supporting a 60-min intensive exercise (i.e., 483 mmol creatine phosphate/whole-body skeletal muscle/min × 20/142 = 68 mmol = 68 mmol × 12.3 kcal/mol × 0.001 × 60 min = 50 kcal). Thus, a small amount of supplemental creatine makes a very significant contribution to energy storage and metabolism in the skeletal muscle of humans to enhance the power and duration of muscular work. In support of this view, dietary supplementation with creatine to young adults decreases oxidative DNA damage and lipid peroxidation induced by a single bout of resistance exercise (Rahimi 2011). Oral administration of creatine phosphate (20 g/day on days 1–3 and 10 g/day for days 4–42) offers a comparable ergogenic effect to that of equal amounts of creatine monohydrate (Peeters et al. 1999).

In the International Society of Sports Nutrition position stand (Kreider et al. 2017), multiple studies have been reviewed to indicate the following. First, there is consistent evidence that oral ingestion of 3 g creatine monophosphate (equivalent to 2.64 g creatine) per day increases intramuscular creatine concentrations. This can be a biochemical basis for improvements in high-intensity exercise performance and training adaptations, as noted previously. Second, creatine supplementation may enhance post-exercise recovery, injury prevention, thermoregulation, and rehabilitation, as well as concussion, spinal cord neuroprotection. Third, creatine supplementation can improve muscular function and enhancing rehabilitation from injuries in subjects with neurodegenerative diseases (e.g., muscular dystrophy, Parkinson’s, Huntington’s disease), diabetes, osteoarthritis, fibromyalgia, aging, brain and heart ischemia, adolescent depression, and pregnancy. Fourth, creatine supplementation can enhance anti-oxidative capacity and reduce oxidant-induced tissue injury. Thus, creatine is one of the most popular and beneficial nutritional ergogenic aids for athletes (Kreider et al. 2017). This conclusion continues to be supported by research findings over the past 2 years. For example, Wang et al. (2018) reported that creatine supplementation combined with exercise training for 4 weeks improved maximal muscular strength and reduced exercise-associated muscle damage in adults. Furthermore, oral consumption of creatine electrolyte improved overall and repeated short duration sprint cycling performance (Crisafulli et al. 2018), as well as anaerobic power and strength in athletes (Hummer et al. 2019).

In addition to its ergogenic effect, creatine has also been used for humans to improve cognitive function (Avgerinos et al. 2018) and reduce traumatic brain injury (Balestrino et al. 2016; Dolan et al. 2019). Furthermore, creatine supplementation ameliorates skeletal muscle dysfunction, enhances muscle functional capacity in patients with chronic obstructive pulmonary disease on long-term O_2_ therapy (De Benedetto et al. 2018), and supports the rehabilitation of tendon overuse injury in athletes (Juhasz et al. 2018). Recently, there have been suggestions that creatine may delay or ameliorate sarcopenia in elderly subjects (Candow et al. 2019a, b), reduce fat accumulation in the liver (da Silva et al. 2017), improve muscle mass or function in cancer patients (Fairman et al. 2019), and maintain the mass and function of ageing bones (Candow et al. 2019a, b).

Growing evidence over the past 30 years shows that dietary supplementation with creatine may provide a safe and effective means in the therapeutic intervention of cancers. For example, creatine has an anti-tumor effect in vitro cell cultures and in vivo various transplanted human and rodent tumors (Kristensen et al. 1999; Lillie et al. 1993; Miller et al. 1993). In addition, Pal et al. (2016) reported that oral administration of creatine (150 mg/kg BW per day) for 10 consecutive days resulted in significant regression of tumor size in Sarcoma-180 tumor-bearing mice and improved the overall survival of the animals. Furthermore, creatine supplementation (1 g/kg BW per day for 21 days) to Walker-256 tumor-bearing rats prevented skeletal muscle atrophy by attenuating systemic inflammation and protein degradation signaling, thereby enhancing their muscle mass and survival (Cella et al. 2019). Similar beneficial effects of creatine supplementation have been obtained for humans with cancers (Fairman et al. 2019).

### Carnosine

#### Tissue distribution of carnosine

In 1900, the Russian biochemist W. Gulewitsch discovered an abundant substance in the skeletal muscle of cattle and named this substance *carnosine* after “caro” or “carnis” (meaning meat in Latin), which was identified in 1918 to be a dipeptide, β-alanyl-l-histidine. Carnosine is characterized by three ionizable groups: the carboxylic group (p*K*a 2.77), the amino group of the β-alanine residue (p*K*a 9.66), and the imidazole ring in histidine (p*K*a 6.83), with the p*K*a of the whole molecule being 8.25 (Tanokura et al. 1976). Thus, at physiological pH (e.g., pH 7.0–7.4), carnosine is present in the zwitterionic form and has a net positive charge.

Concentrations of carnosine in the skeletal muscle of humans without carnosine or β-alanine supplementation range from 5 to 10 mM (Boldyrev et al. 2013), or 16.7–33.3 mmol/kg dry weight of muscle. For example, the soleus and gastrocnemius muscles of adult males contain 8 and 10 mM carnosine, respectively, or 26.7–33.3 mmol/kg dry weight of muscle (Derave et al. 2007). These values are approximately 2, 10 and 20 times greater than those in the skeletal muscle of pigs, rats and mice, respectively (Boldyrev et al. 2013). Note that bodybuilders can have carnosine concentrations as high as 15.3 mM or 51 mmol/kg dry weight, with average values being 13 mM or 43 mmol/kg dry weight (Tallon et al. 2005). The concentrations of carnosine in the olfactory bulb of the brain and the cardiac muscle are comparable to those in skeletal muscle, but those in other tissues (e.g., ~ 0.1 mM in kidneys and adipose tissue) are only 0.1% to 10% of those in skeletal muscle. Based on the report that the mean concentrations of carnosine in the skeletal muscles of women and men are 17.5 and 21.3 mmol/kg dry weight, respectively (Mannion et al. 1992), it can be estimated that a 60-kg women and a 70-kg men have 32 and 45 g carnosine, respectively. In mammals (including humans), about 99% of carnosine is present in skeletal muscle (Sale et al. 2010). Carnosine is negligible or not detectable in the plasma of humans, but is present at low concentrations (2–15 µM) in the plasma of non-primate animals.

Carnosine is more abundant in the white muscle of humans than in their red muscle (Hill et al. 2007). Based on: (1) the percentages of type I and type II fibers in skeletal muscle of adult humans, which are 88% and 12%, respectively, in soleus muscle, and 47% and 53%, respectively, in gastrocnemius muscle (Hill et al. 2007), and (2) the concentrations of carnosine in these two muscles (Derave et al. 2007), it can be calculated (0.47*x* + 0.53*y* = 10 and 0.88*x* + 0.12*y* = 8; *x* and y are carnosine concentration in type I and II muscles, respectively) that type I and type II muscle fibers contain 7.4 and 12.3 mM carnosine, respectively. This means that type II muscle fiber has 66% more carnosine than type I muscle fiber. Such information is important for developing an effective nutritional strategy to enhance muscular carnosine levels and improve muscular strength in athletes and elderly subjects. This is because of the following reasons. First, elite athletes can have up to 80% of type I or type II muscle fiber, depending on the type of their sports, which is a determinant of their success at competition. For example, a sprinter with 80% of white muscle fibers will have a better performance than somebody with only 30% of white muscle fibers (Komi and Karlsson 1978). Second, age-related loss of muscle mass results from a decrease in the total number of both type I and type II fibers and, but secondarily, from a preferential atrophy of type II fibers (Lexell et al. 1988; Roos et al. 1997). Therefore, adequate availability of carnosine in skeletal muscle will be beneficial for healthy ageing.

#### Absorption of carnosine by the small intestine and transport in blood

Dietary carnosine is absorbed by the enterocytes of the small intestine across their apical membrane via peptide transporter-1 (PepT1) (Fig. 1). This is an H^+^-driven process. Inside the enterocytes, a limited amount of carnosine is hydrolyzed by carnosinase-2 into β-alanine and histidine (Sadikali et al. 1975). The intracellular carnosine is exported by peptide/histidine transporters 1 and 2 (PHT1 and PHT2) out of the enterocyte across its basolateral membrane into the lamina propria of the small-intestinal mucosa. PepT1, PHT1 and PHT2 are members of the proton-coupled oligopeptide transporter family (POT-family or SLC15), and have a broad specificity for di- and tri-peptides (including carnosine and its methylated analogs). The main difference between PepT1 and PHTs is that the PHTs can transport l-histidine, in addition to di/tripeptides. Within enterocytes, a small amount of carnosine is hydrolyzed by carnosinase-2 (also known as tissue carnosinase), and nearly all of the ingested carnosine enters the portal circulation (Asatoor et al. 1970). In the blood of humans, carnosine is actively hydrolyzed by carnosinase-1 (also known as serum carnosinase; an enzyme that is synthesized and released from the liver) into β-alanine and histidine, which are then taken up by extra-intestinal cells via specific transporters for beta-amino acids and basic amino acids, respectively. Carnosine in plasma, if any, is transported into extra-intestinal tissues and cells via PepT2 (peptide transporter 2) as well as PHT1 and PHT2. PepT2, which has a broad specificity for di- and tri-peptides as does PepT1, is more widespread than PepT1 in extra-intestinal tissues and cells. Increasing dietary intake of carnosine enhances its concentrations in skeletal muscle, brain and heart (Boldyrev et al. 2013).

Ingestion of 4 g synthetic carnosine alone by adult humans does not affect its concentrations in plasma due to high carnosinase-1 activity in plasma that rapidly hydrolyzes carnosine into β-alanine and histidine, but does increase the urinary output of carnosine (Gardner et al. 1991). Urinary carnosine accounts for 14% of the ingested amount over a 5-h period and peaks at 2 h after the consumption of carnosine. Similarly, within 90 min after the ingestion of 4.5 g carnosine by adult humans, the concentration of carnosine in serum does not change but the concentrations of histidine and β-alanine rapidly increase (Asatoor et al. 1970). This is consistent with the high activity of carnosinase-1 in the plasma of humans to hydrolyze carnosine. Interestingly, consumption of beef by men and women can result in an increase in the concentration of carnosine in their plasma that peaks (145 µM) at 2.5 h after intake and returns to the baseline value at 5.5 h after intake (Park et al. 2005). It is possible that some components in beef [anserine, amino acids (e.g., histidine and β-alanine), and copper] inhibit serum carnosinase (Bellia et al. 2014; Boldyrev et al. 2013), so that the circulating levels of carnosine are substantially elevated. This can directly supply the dipeptide to extra-intestinal tissues (e.g., heart and pancreas) and cells (e.g., red blood cells and immunocytes). In this regard, ingestion of beef as a whole food may be superior to oral administration of synthetic carnosine alone for humans.

#### Synthesis of carnosine in humans

Synthesis of carnosine from β-alanine and histidine is catalyzed by ATP-dependent carnosine synthetase (a cytosolic enzyme) (Wu 2013), which is primarily present in skeletal muscle, heart, and certain brain regions (e.g., the olfactory bulb) (Drozak et al. 2010; Harding and O'Fallon 1979). The sources of β-alanine are diets and endogenous syntheses from the catabolism of aspartate (mainly in bacteria), malonic acid semialdehyde (transamination with glutamate), coenzyme A, pyrimidines, polyamines (Wu 2013). The sources of histidine are diets and the degradation of hemoglobin (which is very rich in histidine), actin, myosin, and other proteins in the body.

Based on the *k*_d_ (fractional turnover rate) value of 0.0133/day for the loss of intramuscular carnosine at the physiological steady state (Spelnikov and Harris 2019), the intramuscular concentrations of carnosine in women and men (17.5 and 21.3 mmol/kg dry weight, respectively), skeletal muscle mass (45% of BW), and its dry matter content (30%), it can be estimated that a 60-kg woman and a 70-kg man synthesizes 427 and 606 mg carnosine per day, respectively. The K_M_ values of this enzyme for β-alanine and histidine are 1.0–2.3 mM (Ng and Marshall 1978) and 16.8 µM (Horinishi et al. 1978), respectively. The value of carnosine synthetase for β-alanine is much greater than or comparable to the intracellular concentration of β-alanine in the brain (0.09 mM) or skeletal muscle (1.0 mM), respectively (Wu 2013). In contrast, the value of carnosine synthetase for histidine is much lower than the intracellular concentration of histidine in the brain (143 µM) and skeletal muscle (404 µM), respectively (Wu 2013). As reported for nitric oxide synthase whose *K*_M_ value for arginine is much greater in cells than that in assay tubes (Wu and Meininger 2000), it is possible that the *K*_M_ of carnosine synthetase for histidine in vivo is much greater than that (i.e., 16.8 µM) reported for the purified enzyme under in vitro assay conditions, due to complex protein–protein and enzyme–substrate interactions in vivo.

Availability of β-alanine primarily limits carnosine synthesis by carnosine synthetase in human skeletal muscle and the olfactory bulb, but adequate provision of histidine in diets is also critical for maximal production of carnosine because histidine is not synthesized de novo (Hill et al. 2007; Sale et al. 2010). For example, supplementation with β-alanine to humans (e.g., 2–6 g/day) dose-dependently increases the concentrations of carnosine in skeletal muscle by 20–80%, but dietary supplementation with 3.5 g histidine/day for 23 days has no effect on intramuscular carnosine concentrations in non-vegetarian adults (Blancquaert et al. 2017; Culbertson et al. 2010). Furthermore, dietary supplementation with 3.2 or 6.4 g β-alanine per day (as multiple doses of 400 or 800 mg) or with l-carnosine (isomolar to 6.4 g β-alanine) per day for 4 weeks augmented intramuscular carnosine concentrations by 42%, 64% and 66% (Harris et al. 2006). These results reveal that histidine is not a limiting factor in carnosine synthesis in adults consuming adequate histidine from animal-source foods. Thus, in non-vegetarian humans, consumption of an equal amount of β-alanine and carnosine effectively increases intramuscular carnosine concentrations to the same extent. It is unknown whether dietary intake of histidine limits carnosine synthesis in vegetarians with or without β-alanine supplementation.

It is noteworthy that dietary supplementation with β-alanine (6 g/day for 23 days) to adults reduces the concentrations of histidine in their plasma and skeletal muscle by 31% and 32%, respectively, possibly due to reduced intestinal absorption of histidine and increased utilization of histidine for carnosine synthesis by skeletal muscle (Blancquaert et al. 2017). Whether this reduction in histidine with β-alanine supplementation has a long-term adverse effect on human health is unknown, but it is prudent to ensure that dietary intake of histidine via supplementation or consumption of histidine-rich foods (e.g., meat) is sufficient. In contrast, oral administration of carnosine increases the concentration of not only β-alanine but also histidine in the plasma of humans (Asatoor et al. 1970), indicating an advantage of the consumption of synthetic carnosine or carnosine-rich foods (e.g., beef) over the consumption of β-alanine alone.

Within a mammalian species, the synthesis of carnosine is affected by multiple factors, including age, sex, muscle fiver type, muscular activity, and diet (Harris et al. 2012). For example, in a study with 9- to 83-year-old humans, Baguet et al. (2012) found that the concentration of carnosine in skeletal muscle increased between 9 and 18 years of age but decreased thereafter with advanced ages. Likewise, the content of carnosine in soleus muscle declines with age between 17- and 47-year-old subjects (Everaert et al. 2011). In adult humans, white muscle fibers contain 30–100% more carnosine than red muscle fibers, and men have 22–82% greater concentrations of carnosine in skeletal muscle than women (Everaert et al. 2011; Mannion et al. 1992). For example, compared to age-matched women, men have 36, 28 and 82% greater concentrations of carnosine in soleus, gastrocnemius and tibialis anterior muscles, respectively (Everaert et al. 2011). It is possible that androgens enhance carnosine synthesis in skeletal muscle, but the circulating levels of testosterone in healthy adult men do not appear to be related to their intramuscular carnosine concentrations (Everaert et al. 2011). Finally, long-term exercise (e.g., 2 days per week for 8 weeks for sprinters) increases intramuscular carnosine levels in male subjects by 113%, which is associated with a 9% increase in the mean power (e.g., during 30-s maximal cycle ergometer sprinting) was significantly increased following training (Suzuki et al. 2004); similar results (a 100% increase in intramuscular carnosine levels) were obtained for resistance-trained bodybuilders (Tallon et al. 2005). Finally, because plant-source foods contain much lower concentrations of β-alanine and histidine (Hou et al. 2019) than animal products (Wu et al. 2016) and because the formation of β-alanine in vegans is limited (Harris et al. 2012), the synthesis of carnosine in vegans is inadequate and its concentrations in soleus and gastrocnemius muscles are 17% and 26%, respectively, lower than those in omnivores who consume some meat that is an excellent source of both β-alanine and histidine (Everaert et al. 2011; Harris et al. 2012).

#### Metabolism of carnosine in humans

Carnosine is hydrolyzed by carnosinase-2 (a Zn^2+^-dependent cytosolic enzyme in tissues) and carnosinase-1 (a Mn^2+^-dependent extracellular enzyme in human blood), but not by proteases or dipeptidases, in humans and animals. Expression of these two isoforms of carnosinase occurs in a tissue-specific manner. Specifically, carnosinase-1 is expressed in the liver, brain, and kidneys but is absent from skeletal muscle and the small intestine (Boldyrev et al. 2013; Everaert et al. 2013). Expression of carnosinase-2 in tissues other than the liver, brain, and kidneys is limited shortly after birth and gradually increases to adult levels during adolescence. Accordingly, the plasma concentrations of carnosine are 5–10 µM in preterm infants, 3–5 µM in term infants, and absent in healthy adults (Asatoor et al. 1970; Valman et al. 1971). In humans, carnosinase-1 that is present in serum at a very high activity is synthesized and secreted mainly by the liver. In contrast, the serum of healthy non-primate mammals (except for the Syrian golden hamster) and birds contains no carnosinase (Boldyrev et al. 2013). Thus, carnosine is stable in the plasma of rats and farm animals but not humans (Yeum et al. 2010). The concentrations of carnosine are 2–15 µM in the plasma of non-primate land animals. In contrast, carnosinase-2 is more widely expressed in mammalian tissues (including skeletal muscle and the small-intestine mucosa of some species), but is absent from the serum or cerebrospinal fluid of mammals or birds (Bellia et al. 2014; Boldyrev et al. 2013; Everaert et al. 2013). The gastric and colonic mucosae of mammals (including humans, pigs, cattle and rats) contain no carnosinase-1 or carnosinase-2 activity.

As noted previously, in humans, dietary carnosine is absorbed intact by the small intestine into the portal circulation, but is rapidly hydrolyzed by serum carnosinase in plasma into β-alanine and histidine; both of the amino acids are reused for carnosine synthesis by skeletal muscle, heart, and the olfactory bulb of the brain (Boldyrev et al. 2013; Harris et al. 2006). In the human skeletal muscle (pH 7.1), which lacks carnosinase-1, the degradation of carnosine by carnosinase-2 is very limited, because its optimal pH 9.5 is much greater than the intracellular pH and its *V*_max_ is 30 times lower than that of carnosinase activity in the human kidneys (Lenney et al. 1985). This explains the relatively high concentration of carnosine in mammalian skeletal muscle. The mean half-life of intramuscular carnosine in adult humans has been estimated to be 52 days (a range of 46–60 days; Spelnikov and Harris 2019). This indicates that carnosine has a relatively high degree of biological stability in the body. Consistent with this view, the turnover rate of carnosine in adult humans at the physiological steady state is 1.33%/day (Spelnikov and Harris 2019). Excess carnosine, β-alanine and histidine are excreted from the body via the urine (Sale et al. 2010). The healthy adult without ingesting carnosine excreted 24 µmol carnosine in 5 h (Gardner et al. 1991). In adult humans consuming 150 g beef or chicken broth, urinary concentrations of carnosine within 7 h after intake were 13- and 15-fold greater, respectively, than the values for no consumption of the meat (Yeum et al. 2010).

#### Physiological functions of carnosine

Carnosine has a functional imidazole ring, which can readily donate hydrogen to free radicals for their conversion into non-radical substances (Kohen et al. 1988). Such an ability of the imidazole ring is enhanced by the β-alanine moiety in carnosine. The major physiological functions of carnosine include: pH-buffering, activation of muscle ATPase to provide energy, metal-ion (copper, zinc and iron) chelation and homeostasis, antioxidant capacity (directly through scavenging ROS and peroxyl radicals and indirectly through chelating metals), and protection against lipid peroxidation, protein oxidation, and the formation of advanced protein glycation (by inhibiting protein carbonylation and glycoxidation) and lipoxidation end products (by suppressing lipid peroxidation) (Barca et al. 2019; Boldyrev et al. 2013; Nelson et al. 2019). The p*K*a value of the imidazole ring of carnosine (6.83) is closer to the intracellular pH in cells than the imidazole ring of free l-histidine (6.04) (Wu 2013). As a positively charged molecule, carnosine can neutralize ATP (~ 5 mM, a negatively charged molecule). Thus, in skeletal muscle, carnosine (5–10 mM) would be a better buffering molecule than free histidine (0.4 mM). Davey (1960) reported that carnosine, together with anserine, accounted for approximately 40% of the pH-buffering capability in the skeletal muscles of rabbits and pigeons. Likewise, Sewell et al. (1992) found that carnosine contributed about 20% of the buffering in type I muscle fibers and up to 46% in type IIb fibers, and these results are consistent with the findings that type I muscle fibers have a lower glycolysis activity and accumulate less lactic acid than type IIb muscle fibers. A similar buffering function of carnosine also applies to the olfactory bulb of the brain, which has only 0.14 mM histidine (Wu 2013). The β-alanine moiety of carnosine contributes to a reduction in its oxidation potential, and carnosine is oxidized at a lower oxidation potential than histidine to remove oxidants (Kohen et al. 1988). Thus, carnosine readily forms a charge-transfer complex with oxygen free radicals (e.g., superoxide radical, hydroxyl radicals, peroxyl radicals, and nitric oxide), non-radical ROS (e.g., peroxynitrite, hypochlorous acid, singlet oxygen, and H_2_O_2_), and deleterious aldehydes (e.g., malondialdehyde and formaldehyde) to confer anti-oxidative effect and protect cell membrane and intracellular organelles (e.g., mitochondria) from damage (Kohen et al. 1988; Pavlov et al. 1993). Besides the anti-ischemic effect of carnosine on the brain and heart, there is evidence that carnosine can maintain the integrity of the DNA molecule, as indicated by studies with telomere (Shao et al. 2004). The latter is a region of repetitive nucleotide sequences at each end of a chromosome, which protects the end of the chromosome from deterioration or from fusion with neighboring chromosomes. Specifically, carnosine can reduce telomere shortening rate possibly by protecting telomeres from damage, thereby contributing to the life-extension effect of carnosine (Shao et al. 2004). Thus, carnosine is beneficial for healthy ageing.

Other physiological functions of carnosine include the regulation of sarcoplasmic reticulum Ca^2+^-release channels and sarcoplasmic reticulum Ca^2+^ homeostasis in skeletal muscle, activation of its phosphorylase activity to promote glycogen breakdown (Johnson et al. 1982), inhibition of the angiotensin converting enzyme, enhancement of nitric oxide availability in endothelial cells, potentiation of cardiac and skeletal muscle contractilities, serving as a neurotransmitter or a neuromodulator, as well as the modulation of excitation–contraction coupling in skeletal muscle and the activity of the sympathetic nerve innervating the muscle (Nagai et al. 2019; Berezhnoy et al. 2019). Carnosine also plays a role in the inhibition of the growth and migration but induction of apoptosis of tumor cells, including human glioblastoma cells as well as colorectal and ovarian carcinoma cells (Hipkiss and Gaunitz 2014; Hsieh et al. 2019; Iovine et al. 2016), as well as the suppression of the release of interleukin-6 by lipopolysaccharides plus interferon-γ-activated macrophages (Caruso et al. 2017). Most recently, carnosine was reported to influence epigenetic regulation of gene expression in mammalian cells via increased histone acetylation (Oppermann et al. 2019). Compared with its constituent amino acids, the formation of carnosine can reduce intracellular osmolarity in the skeletal muscle and olfactory bulb, thereby protecting their integrity and function. In addition, being a positively charged dipeptide that is resistant to peptidases, carnosine does not readily exit cells and can be accumulated inside the cells at high concentrations within physiological ranges. Through its intracellular actions, carnosine confers a vasorelaxing effect to reduce blood pressure (Ririe et al. 2000), stroke, and seizures (Horning et al. 2000), and carnosine is particularly beneficial for ameliorating ageing-related disorders such as cataract and neurological diseases (Cararo et al. 2015; Schön et al. 2019). Thus, carnosine plays an important role in maintaining cell structure and function, as well as whole-body homeostasis and healthy aging, while reducing the risk of oxidative stress-related diseases, such as obesity, diabetes, hypertension, atherosclerosis, Alzheimer’s disease, stroke and seizures.

#### Health benefits of carnosine supplementation

Much research has demonstrated the beneficial effects of dietary supplementation with carnosine on animal models of human diseases (Derave et al. 2019). These effects include decreased plasma glucose and amelioration of diabetic complications (e.g., nephropathy, ocular damage, and retinopathy) in diabetic mice (Lee et al. 2005; Pfister et al. 2011); reduced lipid oxidation and augmented anti-oxidative capacities [e.g., restoring of blood glutathione and basal activities of antioxidant enzymes in aging rats (Aydin et al. 2010a,b; Hipkiss and Brownson 2000)] and in pigs (Ma et al. 2010); amelioration of acetaminophen-induced liver injury (Yan et al. 2009), thioacetamide- or hyperammonemia-induced liver cirrhosis (Aydin et al. 2010a,b; Jamshidzadeh et al. 2017b), and ethanol-induced chronic liver injury in rats (Liu et al. 2008). Dietary supplementation with carnosine also results in decreases in malondialdehyde, oxidative stress (including ischemic oxidative stress and cerebral ischemia), and ethanol-induced protein carbonyls in brains (Berezhnoy et al. 2019; Fedorova et al. 2018); amelioration of neurological disorders, including autism spectrum disorder in children (Chez et al. 2002; Kawahara et al. 2018); protection against cardiovascular injury (Abplanalp et al. 2019; Artioli et al. 2019) and bleomycin-induced lung fibrosis in rats (Cuzzocrea et al. 2007); cardiomyopathy in rats induced by chemotherapeutic agents that cause hydroxyl radical formation and lipid peroxidation (Dursun et al. 2011); ROS-induced renal damage in mice (Fouad et al. 2008); and promotion of wound healing in rodents (Ansurudeen et al. 2012). Through augmenting respiratory burst in neutrophils and their production of ROS, as well as modulating the release of the virulent influenza virus from activated neutrophils, oral administration of carnosine and anserine reduces virus dissemination in humans (Babizhayev and Deyev 2012; Babizhayev et al. 2014).

Extensive animal experiments have laid a strong foundation for human clinical studies, which indicate beneficial effects of supplementation with carnosine (0.5–2 g/day) for 1–6 months on subjects with chronic diseases (Table 2). For example, oral administration of carnosine has been used to ameliorate syndromes in patients with gastric ulcers (Sakae and Yanagisawa 2014), Parkinson disease (Boldyrev et al. 2008), schizophrenia (Chengappa et al. 2012), autistic spectrum disorder (Chez et al. 2002), and ocular diseases (Babizhayev et al. 2001). Carnosine supplementation has also been shown to attenuate elevated levels of glucose, triglycerides, advanced glycation end products, and tumor necrosis factor-α levels in patients with type-2 diabetes (Houjeghani et al. 2018) and in overweight or obese pre-diabetic subjects (de Courten et al. 2016; Liu et al. 2015), enhance cardiac output and improve the quality of life in patients with heart failure (Cicero and Colletti 2017), as well as renal functional integrity and anti-oxidative capacity in pediatric patients with diabetic nephropathy (Elbarbary et al. 2017); ameliorate insulin resistance (Baye et al. 2019), and increase lean-tissue mass in obese or overweight subjects (Liu et al. 2015).

Exercise is associated with increases in Ca^2+^ influx into the myofibers of skeletal muscle (regulating myosin-actin cross bridging and action potential along the muscle fiber membrane) and in the production of ROS and H^+^ by skeletal muscle. Excessive ROS and H^+^ must be removed rapidly, for example, by carnosine to sustain muscular activity and ATP generation. Therefore, dietary supplementation with β-alanine or carnosine improves muscular performance in humans (Matthews et al. 2019). This notion is further substantiated by several lines of evidence. First, increases in carnosine concentrations in soleus (+ 45%) and gastrocnemius (+ 28%) through oral administration of β-alanine (5 g/day) for 7 weeks enhanced rowing performance in athletes (Baguet et al. 2010). Similarly, supplementation with 4–6.4 g β-alanine per day (8 dosing of 0.4 or 0.8 g for each dosing) for 10 weeks to non-vegetarian men (consuming 0.25–0.75 g β-alanine from histidine dipeptides in meat) augmented intramuscular carnosine concentrations by 74% (93% and 57% increase in type I and II muscle fibers, respectively) and improved their high-intensity cycling performance (Hill et al. 2007). Second, dietary supplementation with 2.5 g of a mix of anserine and carnosine (2:1) per day for 13 weeks enhanced cognitive functioning and physical capacity (indicated by the Senior Fitness Test) in the elderly (Szcześniak et al. 2014). Third, oral administration of carnosine (500 mg once daily) for 6 months improved exercise performance (indicated by the 6-min walking test, peak O_2_ consumption, and peak exercise workload), as well as the quality of life in patients with chronic heart failure (Lombardi et al. 2015).

### Anserine

#### Tissue distribution of anserine

After the discovery of carnosine in beef, scientists analyzed this substance in other animal species. In 1929, N. Tolkatschevskaya and D. Ackermann independently identified a carnosine-like compound in goose skeletal muscle to be a dipeptide (methyl carnosine; β-alanyl-1-methyl-l-histidine), which was named *anserine* after the taxonomic name for the goose. This peptide is characterized by three ionizable groups: the carboxylic group (p*K*a 2.64), the amino group of the β-alanine residue (p*K*a 9.49), and the imidazole ring in histidine (p*K*a 7.04), with the p*K*a of the whole molecule being 8.27 (Bertinaria et al. 2011). Thus, at physiological pH (e.g., pH 7.0 to 7.4), anserine is present in the zwitterionic form and has a net positive charge.

Anserine is abundant in the skeletal muscles of birds, certain fish [e.g., salmon, tuna and trout (Boldyrev et al. 2013)], and beef (Wu et al. 2016), but is absent from human tissues (including skeletal muscle, heart and brain) (Mannion et al. 1992). Among the animal kingdom, anserine is the major histidine-containing dipeptide in the skeletal muscles of dog, cat, lion, rabbit, agouti, mouse, kangaroo, wallaby, opossum, cod, smelt, marlin, whiting, croaker, tuna, Japanese char, salmon and trout (Boldyrev et al. 2013). Intramuscular concentrations of anserine in these non-primate species range from 2 mM for opossum to 21 mM for marlin. High concentrations of anserine in the mM range are also present in the brains of the non-primate animals. In contrast, anserine is usually absent from the plasma of healthy adult humans without anserine intake (Everaert et al. 2019) and is present at 2–10 µM in the plasma of non-primate animals depending on species (Boldyrev et al. 2013).

Intramuscular and cardiac concentrations of anserine are affected by a number of factors, including muscle fiber type, muscular contractility, and health status. For example, white muscle fibers contain much more anserine than red muscle fibers, as the concentrations of anserine and carnosine were 2.2- and 2.8-fold higher, respectively, in breast versus thigh muscle (Barbaresi et al. 2019). In rat skeletal muscles (longissimus dorsi and quadriceps femoris), the concentrations of carnosine and anserine decrease by 35–50% during senescence, and are 35–45% lower in hypertensive animals than in normotensive ones (Johnson and Hammer 1992). Similarly, in rat cardiac muscle, the concentrations of total histidine dipeptides decline by 22% during senescence and, are 35% lower in hypertensive animals than in normotensive ones (Johnson and Hammer 1992).

#### Absorption of anserine by the small intestine and transport in blood

In humans, dietary anserine is absorbed by the small intestine, transported in blood, and taken up by extra-intestinal tissues as described previously for dietary carnosine (Fig. 1), except that the rates of catabolism of anserine to β-alanine and 1-methyl-histidine by serum carnosinase (carnosinase-1) in plasma and by carnosinae-2 in non-blood tissues are lower than those for carnosine (Yeum et al. 2010). A study with adult humans has shown that oral administration of 2 g anserine/60 kg BW without or with 19.4 g food increased the concentration of anserine in plasma, which peaked (52 and 40 µM, respectively) at 45 min after consumption and returned to the baseline value at 3 h after consumption (Kubomura et al. 2009). In the absence of food intake, the oral administration of anserine increased the concentration of 1-methyl-histidine in the human plasma, which peaked (125 µM) at 60 min and declined thereafter to ~ 100 µM at 4 h after consumption. When anserine was consumed along with 19.4 g food, because the intestinal absorption of anserine was delayed, the oral administration of anserine increased the concentration of 1-methyl-histidine in the human plasma to the peak value of ~ 100 µM) at 75 min, which remained essentially unchanged at 4 h after consumption. Similar results were obtained for β-alanine (Yeum et al. 2010). In adult humans, the *T*_1/2_ of orally administered anserine in plasma is 1.28 or  1.35 h, respectively, without or with food consumption (Kubomura et al. 2009). Thus, in humans, the circulating anserine is cleared rapidly as is carnosine. Increasing dietary intake of anserine enhances its concentrations in skeletal muscle, brain and heart (Boldyrev et al. 2013).

#### Synthesis of anserine in non-primate animals

Humans and other primates do not synthesize anserine. However, non-primate animals can synthesize anserine from β-alanine and histidine at various rates, depending on species (Boldyrev et al. 2013). The ATP-dependent pathways require anserine synthetase, as well as carnosine synthetase plus carnosine 1-methyltransferase (Wu 2018). The anserine synthetase pathway is a minor one, because 1-methylhistidine is limited in animal tissues. The carnosine *N*-methyltransferase is quantitatively important and physiologically active for anserine synthesis in skeletal muscle. Because carnosine 1-methyltransferase and guanidinoacetate methyltransferase (the enzyme that converts guanidinoacetate into creatine) compete for *S*-adenosylmethionine (Wu 2013), there may be a close metabolic relationship between anserine and creatine syntheses in non-primate animals. Like carnosine, the homeostasis of anserine in skeletal muscle is controlled by the availability of β-alanine or its degradation via transamination (Blancquaert et al. 2017).

#### Metabolism of anserine in humans

Besides using carnosine as a substrate, carnosinase also acts on anserine, but its enzymatic activity is lower for anserine than for carnosine (Boldyrev et al. 2013; Yeum et al. 2010). Thus, anserine is metabolized in humans and other animals as described previously for carnosine, except that serum carnosinase degrades anserine at a lower rate than carnosine and, therefore, oral administration of anserine or anserine-containing food (e.g., beef) can transiently increase the concentration of anserine in the human plasma (Everaert et al. 2019; Yeum et al. et al.^ 2010^). Excess anserine, β-alanine and 1-methyl-histidine are excreted from the body via the urine (Sale et al. 2010). Oral administration of 4, 10 and 20 mg anserine/kg BW to young adults dose-dependently increased its urinary excretion, with peak concentrations of anserine in urine at 90 min after intake (Everaert et al. 2019). Even in subjects consuming 20 mg anserine/kg BW, the peak concentration of anserine in the plasma was only 3 µM, indicating extensive catabolism of this peptide by serum carnosinase. In adult humans consuming 150 g beef or chicken broth, urinary concentrations of anserine within 7 h after intake were 14- and 243-fold greater, respectively, than the values for no consumption of the meat (Yeum et al. 2010).

#### Physiological functions of anserine

Anserine has physiological functions similar to those of carnosine, including H^+^ buffering, antioxidation, modulation of muscle contractility (e.g., excitation and contraction through transmembrane potential maintenance and electromechanical coupling), and regulation of metabolism (Boldyrev et al. 2013; Everaert et al. 2019; Kohen et al. 1988). However, as a methylated metabolite of carnosine, anserine has some biochemical properties that are different from those of carnosine. For example, in contrast to carnosine, anserine does not chelate copper and may not regulate nitric oxide availability in cells (Boldyrev et al. 2013). Also, although anserine and carnosine exhibit an equal anti-oxidative activity (Boldyrev et al. 1988), anserine (1 mM), but not carnosine (1 mM), increased the protein and mRNA levels of heat shock protein-70 in renal tubular cells treated with 25 mM glucose or 20–100 µM hydrogen peroxide (Peters et al. 2018). Furthermore, anserine, but not carnosine, inhibits carnosinase activity (Derave et al. 2019). Thus, anserine may potentiate the action of carnosine in the body.

#### Health benefits of anserine supplementation

Some studies have shown the beneficial effects of dietary supplementation with anserine on animal models of human diseases characterized by oxidative stress. For example, intraperitoneal administration of 0.1 or 1 mg anserine to hyperglycemic rats (250–300 g of body weight) reduced hyperglycemia and plasma glucagon concentrations by suppressing sympathetic nerve activity (Kubomura et al. 2010). Similarly, intravenous administration of anserine every 2 days for 6 days to 12-week-old diabetic db/db mice reduced blood glucose concentration by 20%, vascular permeability by one-third, and urinary proteinuria by 50% (Peters et al. 2018). In addition, Kaneko et al. (2017) reported that oral administration of anserine (10 mg/mouse) to 18-month-old AβPPswe/PSEN1dE9 mice (a model of Alzheimer's disease) for 8 weeks completely recovered the memory deficits, improved pericyte coverage on endothelial cells in the brain, and suppressed glial inflammatory reactions. These results indicate that anserine ameliorates neurovascular dysfunction and improves spatial memory in aged animals. Interestingly, the effects of anserine on the renal function seem to be dependent on its doses via actions on the histaminergic nerve. For example, intravenous administration of 1 µg anserine to urethane-anesthetized rats suppressed the renal sympathetic nerve activity, blood pressure, and heart rate, whereas intravenous administration of 1 mg anserine had the opposite effects (Tanida et al. 2010). Thus, because of its anti-oxidative and vasodilatory actions, dietary supplementation with anserine-rich chicken meat extracts ameliorated carbon tetrachloride-induced hepatic oxidative stress and injury in rats (Peng and Lin 2004), while improving glucose homeostasis and preventing the development of hypertension in stroke-prone spontaneously hypertensive rats (Matsumura et al. 2002).

Besides animal studies, clinical investigations with humans have demonstrated beneficial effects of anserine on their metabolic, neurological, immunological, cardiovascular and renal functions. For example, oral administration of anserine (10 or 100 mg/45 kg BW in 20 ml of tap water) reduced blood glucose levels during an oral glucose tolerance test in humans (Kubomura et al. 2010). Similarly, supplementing anserine plus carnosine (1 g/day, 3:1 ratio, 3 months) to healthy elderly subjects has been shown to preserve verbal episodic memory and brain perfusion (including blood flow in the prefrontal brain (Ding et al. 2018; Hisatsune et al. 2015; Rokicki et al. 2015), attenuate cognitive impairment (Hisatsune et al. 2016; Masuoka et al. 2019), inhibit the production of inflammatory cytokines by peripheral blood mononuclear cells (Katakura et al. 2017), and enhance muscular strength and exercise endurance (Hirohiko et al. 2006). Because avian muscle contains a large amount of anserine as noted previously, much evidence shows that consumption of a popular Asian food known as chicken essence (chicken meat extract) by humans, particularly elderly subjects, has long been a nutritional means to improve human health and prevent chronic diseases. Specifically, oral administration of anserine (0.01–0.6 g/day) to adults can effectively relieve stress and fatigue, ameliorate anxiety, promote post-partum lactation, improve physical capacity and exercise performance, reduce hyperglycemia and hypertension, enhance immunity, prevent ageing-associated neurological (e.g., cognitive and memory) dysfunction and inflammation, and accelerate wound healing (Li et al. 2012; Szcześniak et al. 2014). Furthermore, oral administration of anserine can enhance the ability of humans to defend against infectious disease, as noted previously. Collectively, these results indicate many health benefits of anserine supplementation on multiple systems in humans.

### 4-Hydroxyproline

#### Tissue distribution of 4-hydroxyproline

4-Hydroxyproline (4-hydroxypyrrolidine-2-carboxylic acid; an imino acid; also often referred to as an amino acid) was originally produced from the acid hydrolysates of beef gelatin by E. Fischer in 1902. Subsequently, 4-hydroxyproline was found to be an abundant constituent of collagen and elastin. Studies in the 1960s revealed that 4-hydroxy-l-proline is derived from the post-translational hydroxylation of l-proline in proteins (primarily collagen). Humans and animals also contain 3-hydroxyproline. In collagen and physiological fluid, the ratio of 4-hydroxyproline to 3-hydroxyproline is 100:1, and these two imino acids exist in the *trans* form (Wu et al. 2019).

The healthy 70-kg adult human contains 15.1% protein (or 10.6 kg protein; Wang et al. 2003), including 3.72 kg collagens (Meléndez-Hevia et al. 2009). In humans and animals, collagen is the most abundant protein comprising 30% and 35% of body protein at birth and in adult life, respectively (Wu et al. 2011). Collagen consists of 13.0 proline, 9.01 4-hydroxyproline, 0.09 3-hydroxyproline, and 33 glycine residues per 100 amino acid residues (or 13.3 g proline, 10.73 g 4-hydroxyproline, 0.11 g 3-hydroxyproline, and 25.8 g glycine residues per 100 g collagen). Thus, proline plus hydroxyproline accounts for approximately 12.5% (g/g) of proteins in the body, with the ratio of proline to 4-hydroxyproline being 2.25:1 (Devlin 1992). In the Gly–X–Y repeat of collagen, proline is in the X or Y position but 4-hydroxyproline occurs only in the Y position.

4-Hydroxyproline is abundant in mammalian milk and plasma (mM range) in the peptide form [primarily glycyl-prolyl-4-hydroxyproline (Gly-Pro-Hyp)], and free 4-hydroxyproline is also present in these physiological fluids (µM range). For example, the concentrations of Gly-Pro-Hyp in the plasma of 7- to 21-day-old pigs and sow’s milk range from 6 to 10 mM and from 2 to 3 mM, respectively (Wu et al. 2019). For comparison, the concentrations of free 4-hydroxyproline in the plasma of mammals are 10 to 125 µM [e.g., 10–15 µM, adult humans (Knight et al. 2006) and 109 µM, 7-day-old pigs (Wu et al. 2019)], depending on diet, species, and age, as well as physiological (e.g., stress) and pathological (cancer) conditions.

#### Absorption of 4-hydroxyproline by the small intestine and transport in blood

4-Hydroxyproline-containing proteins (primarily collagens) are hydrolyzed by proteases in the luminal fluids of the stomach (pepsin) and small intestine (trypsin, chymotrypsin, elastase, carboxypeptidases A and B, and aminopeptidase) to yield peptides and free amino acids (Wu et al. 2011). The oligopeptides are further hydrolyzed by peptidases to tripeptides, dipeptides, and amino acids. The mucosa of the small intestine secretes proline peptidase that specifically hydrolyzes proline-containing dipeptides (Sjostrom et al. 1973). Some prolyl-4-hydroxyproline (Pro-Hyp, a product of Gly-Pro-Hyp degradation) is hydrolyzed to proline and 4-hydroxyproline by intestinal prolidase. This multifunctional enzyme possesses a unique ability to degrade imidodipeptides (X-Pro or X-Hyp) in which a proline or 4-hydroxyproline residue is located at the C-terminal end (Lupi et al. 2008). Thus, hydroxyproline-containing dipeptides and tripeptides in the lumen of the small intestine are transported intact into enterocytes (absorptive epithelial cells) by H^+^ gradient-driven peptide transporters (Fig. 1). In contrast, free proline and 4-hydroxyproline in the lumen are taken up into the cells primarily by the Na^+^-dependent system IMINO transporter and the system NBB transporter (present on the brush border for the transport of neutral amino acids), as well as the Na^+^-independent system L transporter (Brandsch 2006). Inside the enterocyte, some of the 4-hydroxyproline-containing tri- and di-peptides undergo hydrolysis by peptidases and/or cytosolic prolidase to form free amino acids, including 4-hydroxyproline. The latter is metabolized locally to form glycine (Wu et al. 2019). The remaining di- and tri-peptides exist the enterocyte across its basolateral membrane into the lamina propria of the intestinal mucosa via PHT1/2 (Fig. 1). In contrast, proline and 4-hydroxyproline exit the enterocyte across its basolateral membrane into the lamina propria of the intestinal mucosa via specific transporters. Some (about 40%) of the absorbed free proline and hydroxyproline are degraded by the intestinal mucosa and the remaining (about 60%) enters the portal circulation (Li and Wu 2018). Studies with rats have shown that free 4-hydroxyproline and 4-hydroxyproline-containing di- and tri-peptides in the portal vein account for approximately 30% and 70% of the total food-derived 4-hydroxyproline measured in the plasma (Osawa et al. 2018). Note that Gly-Pro-Hyp [a major small peptide from dietary collagen hydrolysates (Yazaki et al. 2017)] in the plasma was not determined in the published study (Osawa et al. 2018). These results indicate that small peptides from collagen hydrolysates are absorbed by the small intestine into the portal circulation predominantly as tri- and di-peptides.

Most of the diet-derived 4-hydroxyproline-containing di- and tri-peptides are not hydrolyzed by the small intestine during the first pass and, therefore, enter the portal circulation (Shigemura et al. 2011). The absolute bioavailability of collagen hydrolysates to the small intestine and extra-intestinal tissues in various amino acid and peptide forms is about 90–95% in humans and animals, depending on species and age. In humans, ingestion of collagen hydrolysates increases the concentrations of at least 11 Hyp-containing peptides (Pro–Hyp, Hyp–Gly, Ala–Hyp, Ile–Hyp, Leu–Hyp, Phe–Hyp, Glu–Hyp, Pro–Hyp–Gly, Gly–Pro–Hyp, Ala–Hyp–Gly, and Ser–Hyp–Gly) in plasma (Sato et al. 2019). A majority of studies have shown that Pro-Hyp and Hyp-Gly, which are resistant to peptidases in the human plasma, are the most (about 50%) and second most abundant food-derived 4-hydroxyproline-containing peptides in the plasma of healthy adult humans, rats and mice after consuming collagen hydrolysates (Osawa et al. 2018; Sato et al. 2019; Shigemura et al. 2014). The small peptides are cleared rapidly from the blood. The maximum concentration of food-derived 4-hydroxyproline-containing peptides in the plasma of individuals consuming collagen hydrolysates (385 mg/kg BW) is 140 µM (the mean value) at 2 h after administration, with the maximum concentration of free 4-hydroxyproline in the plasma being 120 µM (the mean value) at 1–2 h after administration (Ohara et al. 2007). Interestingly, Gly–Pro–Hyp and Pro–Hyp are the most and second most abundant small 4-hydroxyproline-containing peptides in the plasma of mice, whereas Ala–Hyp, Gly–Pro–Hyp and Pro–Hyp are the most, second most, and third most abundant food-derived 4-hydroxyproline-containing peptides in the plasma of adult men and women, at 1–2 h after the animals and human subjects consumed high amounts of tripeptide (Gly–X–X)-containing collagen hydrolysates (0.9–1.8 g/kg BW for mice and 0.3 g/kg BW for humans) (Yazaki et al. 2017). Thus, the abundance of 4-hydroxyproline-containing di- and tri-peptides in the plasma is affected by collagen hydrolysate preparations.

Proline, 4-hydroxyproline, and their small peptides are transported in blood in the free form. They are rapidly taken up by extra-intestinal tissues and cells [e.g., kidneys, skeletal muscle, skin, heart, and immunocytes (Yazaki et al. 2017)] via imino or peptide transporters (Wu 2013). Inside these tissues and cells, the 4-hydroxyproline-containing di- and tri-peptides are hydrolyzed by peptidases and/or prolidase to yield their constituent imino acids (4-hydroxyproline or proline) and glycine. Increasing dietary intake of 4-hydroxyproline in the free or peptide form enhances the concentrations of 4-hydroxyproline in plasma and tissues, such as the skeletal muscle, colon, joints, and skin (Ji et al. 2018; Yazaki et al. 2017).

#### Synthesis of 4-hydroxyproline in humans

In fibroblasts, 4-hydroxyproline is formed from the post-translational hydroxylation of proline residues in proteins, primarily collagen (Li and Wu 2018), as noted previously. Specifically, proline is incorporated into protein through the intracellular pathway of protein synthesis. Thereafter, some proline residues are hydroxylated in the endoplasmic reticulum by prolyl 4-hydroxylase in the presence of oxygen, ascorbic acid, α-ketoglutarate, and Fe^2+^ to form 4-hydroxyprolyl residues (Gorres and Raines 2010). Hydrolysis of the protein containing hydroxylated proline residues releases 4-hydroxyproline as a free imino acid. Other prolyl 4-hydroxylases, including hypoxia-inducible transcription factor α, can convert a limited number of proline residues to 4-hydroxyproline residues in non-collagen proteins (Myllyharju and Koivunen 2013).

#### Metabolism of 4-hydroxyproline in humans

Relatively little is known about 4-hydroxyproline catabolism in humans or animals. However, there is evidence that the kidneys of adult rats can rapidly take up extracellular 4-hydroxyproline and then convert it into glycine via 4-hydroxyproline oxidase (Lowry et al. 1985). Other extra-intestinal tissues (e.g., liver, skeletal muscle, and skin) also synthesize glycine from 4-hydroxyproline (Hu et al. 2017). The half-lives of free hydroxyproline (45 to 80 min) and its di- and tri-peptides (25–100 min) in blood are relatively short and vary with the species and age of mammals. For example, we found the half-lives of free 4-hydroxyproline in the blood of 8- and 160-day-old pigs are 46.2 ± 2.8 and 60.5 ± 3.3 min (mean ± SEM, *n* = 6), respectively, as determined by intravenous administration of 4-hydroxyproline (50 mg/kg BW) and the single exponential model of its pharmacokinetics (Wu et al. 2007). The half-lives of Gly–Pro–Hyp and Pro–Hyp in the blood of adult humans are estimated to be about 50 and 100 min, respectively, but are about 25 min in mice (Yazaki et al. 2017). Similarly, based on the work of Kusubata et al. (2015), the half-life of Pro–Hyp in the blood of mice is estimated to be about 25 min.

The metabolic pathway for glycine synthesis from 4-hydroxyproline, with glyoxylate as the most immediate intermediate (Wu et al. 2019). Interestingly, the estimate rate of whole-body glycine synthesis in the 70-kg healthy human adult (8.16 g/day) is comparable to the measured rate of 10.1 g/day (Gibson et al. 2002) and 8.09 g/day (Yu et al. 1985) in the healthy human adult consuming 0.75 and 1.5 g protein/day, respectively. A small fraction of the glyoxylate is converted into oxalate and glycolate in the liver (Knight et al. 2006). In adult humans, about 95% of the collagen-derived 4-hydroxyproline may be converted into glycine, and the remaining 5% oxidized to oxalate and glycolate (Knight et al. 2006). The amounts of 4-hydroxyproline, oxalate and glycolate excreted daily in the urine of adult humans are 3–4, 1–3, and 10–20 mg, respectively. We estimated that milk-borne and collagen-derived 4-hydroxyproline contribute to 14.4% and 31.1%, respectively, of glycine needed by the young pig, whereas milk-borne glycine and non-hydroxyproline amino acids contribute to 24.0% and 30.5%, respectively, of glycine needed by the neonate (Wu et al. 2019). In adult humans, 4-hydroxyproline may contribute to about 60% of total glycine requirement (Table 3). 4-Hydroxyproline oxidase is the rate-controlling enzyme in the conversion of 4-hydroxyproline into glycine (Wu et al. 2011) and is induced by cortisol (Phang et al. 2010). Thus, the circulating levels of hydroxylproline are reduced in animals with fatigue (caused by deprivation of rest and sleep; a physical stress condition) or oxidative stress (Kenéz et al. 2018; Kume et al. 2015).

**Table 3:** Metabolic needs for glycine and its dietary provision in the 70-kg healthy adult human

Variable	Amount g/day
Dietary protein intake^a^	52.5
Bioavailability of dietary glycine	1.42
Glycine intake from diet^a^	1.58
Dietary glycine not digested (10%)	0.16
Digestible glycine intake from diet	1.42
Needs for glycine	10.1
Heme synthesis^b^	0.25
Creatine synthesis^b^	1.00
Purine synthesis^b^	0.25
Glutathione synthesis^c^	0.57
Net serine synthesis^a,d^	1.36
Bile salt synthesis^c^	0.06
Hippurate synthesis^c^	0.54
Irreversible loss through oxidation to CO_2_^a^	5.03
Urinary glycine loss^c^	0.11
Sweat and dermal glycine loss^c^	0.08
Ileal endogenous glycine loss^e^	0.66
Colonic endogenous glycine loss^e^	0.15
Glycine synthesis (calculated)^f^	8.16
From serine^c^	2.54
From dietary choline^c^	0.107
From threnoine^c^	0.022
From endogenous sarcosine^c^	0.142
From carnitine^c^	0.006
From endogenous 4-hydroxyproline (Hyp)^g^	5.34
Glycine synthesis (measured)^g^	9.10
Dietary glycine intake meeting glycine needs	14%
Contribution of Hyp to whole-body glycine synthesis	59%

^a^Gibson et al. (2002). Adult humans consume 0.75 g protein/kg BW/day

^b^Yu et al. (1985). This value refers to the healthy adult consuming 1.5 g protein/day

^c^Meléndez-Hevia et al. (2009)

^d^The value is estimated from the plasma flux of glycine (22.6 g/day in the 70-kg healthy adult; Ginson et al. 2002) and the net conversion of plasma glycine into serine (i.e., 6% of plasma glycine flux; Butterworth et al. 1958)

^e^Starck et al. (2018)

^f^The average value of 10.1 g/day for the healthy adult consuming 0.75 g protein/day (Gibson et al. 2002) and 8.09 g/day for the healthy adult consuming 1.5 g protein/day (Yu et al. 1985)

^g^The value was calculated on the basis of the following: (1) the rate of degradation of mature collagens in the extracellular matrix is equal to the rate of net synthesis of collagen (i.e., the rate of collagen secreted from fibroblasts to the extracellular matrix; 96.5 g/day) in the healthy adult human (Meléndez-Hevia et al. 2009); (2) the content of 4-hydroxyproline in collagen is 10.73 g/100 g collagen; Wu et al. 2011); and (3) 90% of collagen-derived 4-hydroxyproline is catabolized to form glycine in the healthy adult human (Knight et al. 2006); namely, 96.5 × 10.73/100 × 0.90 × 75.07/131.13 = 5.34 g/day

#### Physiological functions of 4-hydroxyproline

As a key component of collagen, proline and 4-hydroxyproline permit the sharp twisting of the collagen helix. This allows for establishing and maintaining the rigid structure of the collagen molecule in connective tissues, particularly skin, tendon, cartilage, bone, blood vessels, and the basement membrane (e.g., the intestinal lamina propria; a thin, fibrous, extracellular matrix of tissue that separates an epithelium from its underlying tissue), as well as protecting other tissues in the body (Phang et al. 2010). In addition, the presence of 4-hydroxyproline in the Gly–X–Y collagen peptides reduces chemotaxis and blocks apoptosis in neutrophils, while the hydroxylation of two proline residues in hypoxia-inducible factor-α to form 4-hydroxyproline under normoxic oxygen conditions triggers the proteasomal degradation of the protein to regulate its abundance (Wu et al. 2019).

Multiple tissues in humans and many animals synthesize glycine from 4-hydroxyproline, as noted previously. This metabolic pathway is not dependent on folate (whose provision in diets is quantitatively low relative to glycine requirement) and, therefore allows for the provision of glycine from the inter-organ metabolism of amino acids (e.g., arginine, glutamine, glutamate, ornithine and proline) other than the tetrahydrofolate-dependent hydroxymethyl transferation of serine. The synthesis of glycine from 4-hydroxyproline plays an important role in maintaining the homeostasis of glycine [an amino acid with enormous nutritional and physiological importance, including heme formation and anti-oxidative reactions (Wu 2013)], as typical diets can meet at most 20% and 14% of daily glycine needs in milk-fed neonates (Hou et al. 2015) and adult humans (Table 3). In addition, glycine is a major limiting factor for the syntheses of glutathione (the most abundant low-molecular-weight antioxidant in cells) (McCarty et al. 2018), as well as bile salts, purines, heme and creatine in the body (Wu 2013).

Growing evidence shows that 4-hydroxyproline can scavenge oxidants (Phang et al. 2010), suppress NF-*k*B activation (Ji et al. 2018), regulate the intracellular redox state, and stimulate the expression of anti-oxidative enzymes in cells (Wu et al. 2019). Furthermore, 4-hydroxyproline inhibits the production of hydroxyl radical via the Fenton reaction possibly through: (a) formation of coordinate bonds with iron, (b) sequestration of Fe^2+^, and (c) interactions with intermediates of the reaction via hydrophobic hydration (Milić et al. 2015). In vitro anti-oxidative effects (e.g., scavenging of radicals and inhibition of lipid peroxidation) have also been demonstrated for the enzymatic hydrolysates of porcine collagen (Ao and Li 2012). Similarly, 4-hydroxyproline-containing collagen hydrolysates reduce inflammation and promote collage synthesis in dermal fibroblasts (Offengenden et al. 2018), while enhancing bone density and strength (Watanabe-Kamiyama et al. 2010). Finally, as an activator of the apoptotic cascade, oxidation of 4-hydroxyproline by 4-hydroxyproline oxidase to ROS  can inhibit the growth of cancer cells and promote their death (Cooper et al. 2008), thereby inhibiting tumorigenesis. Furthermore, through the action of ROS to kill pathogenic bacteria, fungi, parasites, and viruses (Vatansever et al. 2013), 4-hydroxyproline can enhance the ability of humans against infectious diseases.  Thus, in humans and animals, the generation of 4-hydroxyproline is enhanced in response to oxidative stress as an adaptation mechanism for defense and survival (Wu et al. 2019).

#### Health benefits of 4-hydroxyproline supplementation

Research on the health benefits of 4-hydroxyproine has focused on its role in improving joint, skin and bone health (Moskowitz 2000; Zhang et al. 2011a) and in preventing gut inflammation in animal models. For example, Pro-Hyp stimulates the growth and migration of fibroblasts in the mouse skin (Shigemura et al. 2009) and, therefore, would have important implication for maintaining dermal hydration and accelerating wound healing (Li and Wu 2018). Furthermore, Pro–Hyp inhibits the differentiation of chondrocytes into mineralized cells to maintain their normal physiological activity, while increasing glycosamino-glycan content in the extracellular matrix (Nakatani et al. 2009). Consistent with this view, dietary supplementation with 0.3% Pro-Hyp reduced the breakdown of articular cartilage and ameliorates osteoarthritis in C57BL/6J mice (Nakatani et al. 2009). Likewise, dietary supplementation with 4-hydroxyproline-containing collagen peptides (0.2 g/kg BW per day) to mice attenuated UV-B-induced decreases in skin hydration and soluble type I collagen, hyperplasia of the epidermis, and skin damage (Tanaka et al. 2009). Furthermore, oral administration of the collagen hydrolysates enhanced bone density (Wu et al. 2004) and femur fracture healing in rats (Tsuruoka et al. 2007), as well as cutaneous wound healing in diabetic rats (Zhang et al. 2011b) and injured nondiabetic rats (Zhang et al. 2011c).

There is growing interest in the role of 4-hydroxyproline in intestinal health. Ji et al. (2018) reported that oral administration of 4-hydroxyproine (1% in drinking water) attenuated dextran sulfate sodium (DSS)-induced colitis (characterized by the infiltration of mononuclear cells and colonic mucosal damage) in mice through inhibiting the NF-κB signaling and oxidative stress. Likewise, intragastric administration of a 4-hydroxyproline-containing dipeptide (Pro–Hyp) to DSS-challenged mice at the dose of 227 mg/kg BW per day (1 mmol or 131 mg 4-hydroyproline/kg BW per day) for 8 days mitigated DSS-induced colitis (Zhu et al. 2018). Similarly, intraperitoneal administration of a synthetic 4-hydroxyproline-containing polypeptide [(Gly–Pro–Hyp)_10_] ameliorated clinical symptoms and histopathological colonic changes in mice with acute DSS colitis (Heimesaat et al. 2012). Because colitis increases the risk for colon cancer (the third most common type of cancer diagnosed in the US) (Healy et al. 2019), dietary supplementation with 4-hydroxyproline may help to prevent and treat this disease. Future studies are warranted to test this novel and important hypothesis.

Clinical studies with humans have shown tremendous benefits of dietary supplementation with 4-hydroxyproline-containing peptides on skin, joint and bone health. For example, in a randomized double-blind, placebo-controlled trial involving 35–55 year-old women, oral administration of 2.5 or 5.0 g of 4-hydroxyproline-containing collagen peptide once daily for 8 weeks enhanced skin elasticity (Proksch et al. 2014). In addition, daily consumption of collagen hydrolysates enhances the moisture content in the epidermis of women during winter (Matsumoto et al. 2006) and ameliorates joint pain in subjects with knee osteoarthritis (Deal and Moskowitz 1999). Likewise, dietary supplementation with collagen hydrolysates (10 g/day) to 40–59 year-old women for 8 weeks increased collagen density in the dermis, while decreasing the fragmentation of the dermal collagen network, with the effects occurring at Week 4 and persisting through Week 12 (Asserin et al. 2015). Furthermore, daily oral administration of 5 g of a collagen-hydrolysate food product (containing 0.1 or 2 g Pro–Hyp and Hyp–Gly per kg product) by 35–55 year-old women for 8 weeks resulted in dose-dependent improvements in facial skin conditions, such as skin moisture, elasticity, wrinkles, and roughness (Inoue et al. 2016). Of particular interest, 12-month daily oral administration of 5 g collagen hydrolysates (containing 4-hydroxyproline) augmented bone mineral density in postmenopausal women (König et al. 2018), suggesting their role in preventing osteoporosis. Similarly, dietary supplementation with 4-hydroxyproline-containing collagen hydrolysates (10 g/day) in combination with calcitonin for 24 weeks has shown positive effects on mitigating osteoporosis in postmenopausal women (Adam et al. 1996). The beneficial effects of collagen peptides on humans can result from not only 4-hydroxyproline but also proline and glycine. The latter two amino acids are also abundant in beef (Wu et al. 2016).

## Physiological and dietary requirements for taurine, creatine, carnosine, anserine, and 4-hydroxyproline

Humans and animals have physiological requirements for taurine, creatine and carnosine (Wu 2013). At present, dietary requirements of taurine, creatine, carnosine, anserine, and 4-hydroxyproline have not been established for humans or animals. The Institute of Medicine (IOM 2006) recommended the dietary requirements of infants, children and adults for the so-called nutritionally essential indispensable) amino acids that are not synthesized by their tissues, but did not recommend dietary requirements for the so-called nutritionally nonessential dispensable) amino acids (e.g., arginine and taurine) that are synthesized de novo by their tissues. This does not necessarily mean that humans have no dietary requirements for taurine, creatine or carnosine.

Historically, growth or nitrogen balance was the sole criterion to define whether or not an amino acid or a nitrogenous substance was nutritionally essential for animals (Hou et al. 2015). Similarly, the criterion for assessing nutritional essentiality of amino acids for humans was a short-term (8-day) nitrogen balance in healthy young adults (Rose 1957). Whether an amino acid was classified as nutritionally essential or nonessential is only the matter of definition, and should not reflect the physiological needs of animals or humans for the nutrient (Hou and Wu 2017, 2018). It must be recognized that short-term nitrogen balance studies are not always sensitive to identify the essentiality of all amino acids for the organisms. For example, nitrogen balance was maintained in normal adult humans fed histidine-free diets for 8 days (Rose 1957), but there are no metabolic pathways for histidine synthesis in mammalian cells (Wu 2013). A negative nitrogen balance did not occur in healthy adults during a short period (e.g., 8 days) of consuming a histidine-free diet because the hydrolysis of histidine-rich hemoglobin in blood and of carnosine in skeletal muscle provides histidine for protein synthesis in tissues at the expense of health. Furthermore, it has been known over 75 years that feeding an arginine-deficient diet to adult men for 9 days did not result in a negative nitrogen balance, but decreased sperm counts by  ~ 90% and increased the percentage of non-motile sperm  ~ 10 fold (Holt and Albanese 1944). This striking observation underlines a crucial role of arginine in spermatogenesis and argues that functional needs should be a criterion for the classification of arginine and any other amino acid as an essential or nonessential nutrient in diets (Wu et al. 2000, 2013). Of note, the Institute of Medicine (2006) stated that dietary arginine was not required by healthy adults because arginine is synthesized via the hepatic urea cycle. However, there is no net synthesis of arginine in the mammalian liver via the hepatic urea cycle (Wu and Morris 1998). This demonstrates the importance of ensuring that recommendations for dietary amino acid requirements be based on up-to-date knowledge of new developments in the field of amino acid metabolism.

Taurine, creatine, and carnosine must be provided in diets for individuals who do not synthesize these nutrients due to inborn errors of metabolism (Wu 2013). By the criterion of maintaining the integrity of tissues (e.g., eyes, heart and skeletal muscle), taurine is clearly a nutritionally essential amino acid for humans without inborn errors of metabolism, particularly the young, as noted previously. Based on functional needs, creatine can be classified as a conditionally essential nutrient for humans, particularly athlete vegans who may not have adequate intakes of arginine, glycine and methionine. Because glycine intake from typical diets meets < 20% of physiological needs and the endogenous synthesis of glycine may be insufficient for the optimal requirements of healthy adults (Table 3), dietary supplementation is expected to beneficial for their optimal health, particularly under stress conditions and during ageing (Wu 2016). Likewise, while a lack of carnosine or anserine in diets is not fatal, consumption of these two dipeptides can improve human health, especially neurological,  immunological, retinal, cardiac and muscular functions. Furthermore, ingestion of 4-hydroxyproline (an antioxidant and a precursor of glycine) may protect the gastrointestine from oxidative stress and inflammation, thereby possibly reducing risk for stomach, colon and breast cancers as well as possibly other types of cancer.

Physiological requirement of a healthy 70-kg adult for taurine may be 75 mg/day or 1.07 mg/kg BW per day based on its urinary excretion of 72 mg/day (Laidlaw et al. 1988). Because of their growth and development, infants and children likely have greater physiological requirements for taurine per kg BW than do the human adults. Likewise, based on the urinary excretion of creatinine, a 70-kg healthy adult needs 1.7 g creatine/day to meet physiological needs. Dietary provision of creatine can spare the use of amino acids for protein synthesis and other important pathways such as the production of polyamines and nitric oxide to improve the function of multiple systems. Thus, dietary intake of creatine is beneficial for metabolic health. Based on the whole-body turnover rate of 1.33%/day for carnosine and the total amount of carnosine (45.5 g) in a 70-kg man (Spelnikov and Harris 2019), it can be estimated that the physiological need for this dipeptide by a 70-kg subject is 606 mg/day. Anserine is not a physiological constituent in humans without consuming this dipeptide and, therefore, humans do not appear to have a basal physiological need for it. However, oral administration anserine in the amount of 156 mg/day can enhance whole-body insulin sensitivity in subjects with hyperglycemia (Kubomura et al. 2010). Thus, dietary intake of anserine can improve human health. Finally, because 4-hydroxyproline is the post-translational product of protein-bound proline, humans have no dietary requirements for 4-hydroxyproline. However, dietary 4-hydroxyproline can augment intestinal anti-oxidative capacity and endogenous glycine synthesis. Based on studies with mice receiving intragastric administration of 227 mg prolyl-hydroxyproline/kg BW per day (1 mmol or 131 mg 4-hydroyproline/kg BW per day) (Zhu et al. 2018) and the mouse to human conversion factor of 0.08 (FDA 2005), we suggest that dietary intake of 18.2 mg 4-hydroxyproline/kg BW per day for adult humans may be beneficial for improving intestinal health and preventing colitis.

Beef is a rich source of high-quality protein as well as highly bioavailable vitamin B_12_, zinc, and iron (Wu et al. 2014). Notably, compared with an isocaloric plant food-based snack composed of corn, beans and greens, daily supplementation with a 60-g beef (12.8 g protein)-based snack (250 kcal) to plant-source diets containing little or no animal-source protein (mean = 3.1 g animal-source protein/day) for 21 months prevents nutritional growth stunting, while enhancing skeletal muscle mass and improving immune function in 6- to 14-year-old children (Grillenberger et al. 2003; Neumann et al. 2007). An important concept emerging from this review is that beef is an abundant source of taurine, creatine, carnosine, anserine, and 4-hydroxyproline as physiologically important nutrients for infants, children, and adults to maintain their health and prevent chronic diseases. For example, 30 g of dried beef can provide 80.4 mg taurine, which can meet 107% of daily taurine requirement of the 70-kg adult (Table 4). Similarly, 30 g of dried beef can provide 608 mg carnosine, which can meet 100% of daily carnosine requirement of the 70-kg adult (Table 4). Likewise, meat provides a large amount of creatine, and the consumption of beef can substantially contribute to physiological needs of humans for this nutrient (Table 4). Finally, beef contains abundant anserine and 4-hydroxyproline to improve the health and well-being of the young, adult, and ageing populations. Thus, beef is a functional food for humans.

**Table 4:** Estimated physiological requirements of the 70-kg adult human for taurine, creatine, carnosine, anserine, and 4-hydroxyproline for optimal health

Nutrient	Physiological requirement		Provision of nutrient from 30-g dry weight of beef (mg)	Beef (30 g dry weight) meeting daily physiological requirement (%)
	mg/day	mg/kg BW per day		
Taurine	75	1.07	80.4	107
Glycine	10,100	144	980^a^	10
Creatine	1,700	24.3	303	18
Carnosine	606	8.66	608	100
Anserine	–	–	96.9	–
4-Hydroxyproline	Unknown	Unknown	52.4	Unknown

“–” Denotes no basal physiological requirement

^a^62% of the daily glycine intake by the healthy adult consuming 0.75 g protein/day (Gibson et al. 2002)

## Perspectives

**Fig. 3:**
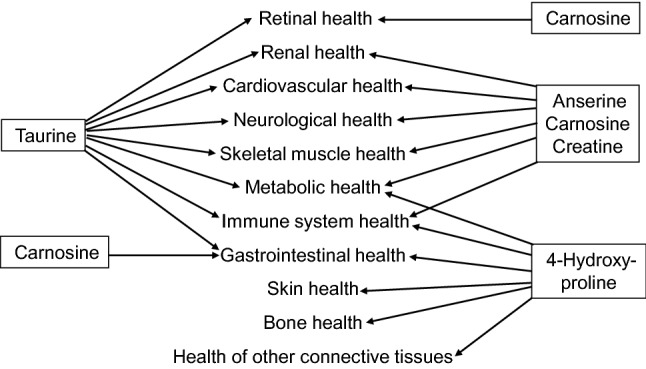
Major functions of dietary taurine, creatine, carnosine, anserine and 4-hydroxyproline on improving the health of multiple systems in humans. These beneficial effects of the nutrients are summarized on the basis of available evidence in the current literatrure. Some of the effects are tissue- and nutrient-specific. However, because all the systems of the body are integrated, the health of one system can affect that of other systems

There are concerns that consumption of red meat increases risks for chronic diseases or metabolic disorders in humans, including obesity, type II diabetes mellitus, cardiovascular disease (the number one killer in the developed nations), ageing-related dysfunction and atrophy of organs (particularly, the brain and skeletal muscle), and cancers (Willet et al. 2019). However, there is no direct evidence to support this view (Johnston et al. 2019; Leroy and Cofnas 2019). Compelling evidence shows that taurine, creatine, carnosine, anserine, and 4-hydroxyproline, which are all abundant in red meat (e.g., beef, lamb and pork), play an important role in inhibiting oxidative stress (a common trigger of chronic diseases) and inflammation, ameliorating tissue (e.g., brain, heart, skeletal muscle, kidney, liver, gut, eye, and connective tissue) injury, improving metabolic profiles and the health of multiple systems, and enhancing immunity in animals and humans (Fig. 3). These nutrients may help to kill pathogenic bacteria, fungi, parasites, and viruses (including coronavirus) through enhancing the metabolism and functions of monocytes, macrophages and other cells of the immune system. Of note, taurine, creatine, carnosine, anserine can inhibit tumorigenesis and promote the apoptosis of tumor (e.g., colon cancer) cells, whereas 4-hydroxyproline prevents colitis and thus possibly reduces risk for colon cancers. Thus, red meat is a functional food for optimizing human growth, development and health. As part of a healthy diet, regular consumption of red meat may actually prevent oxidative stress, thereby reducing risks for chronic diseases in humans. Future studies with animal models and human subjects are warranted to test this hypothesis.

## Conclusion

Dietary taurine, creatine, carnosine, anserine, and 4-hydroxyproline (which are all abundant in beef) play an important role in inhibiting oxidative stress (a common trigger of chronic diseases) and inflammation, ameliorating tissue (e.g., brain, heart, skeletal muscle, kidney, liver, and gut) injury, and improving metabolic profiles in animals and humans. Such a comprehensive update, along with recent advances in amino acid nutrition and physiology, will be highly informative to educate the public and policy makers about animal-source foods (e.g., beef) for human consumption. The health and ergogenic effects of dietary taurine, creatine, carnosine, anserine, and 4-hydroxyproline are expected to reverse the drastic decline in consumption of red meat (e.g., beef) in the U.S. due to an inadequate understanding of animal-source foods to provide functional amino acids, peptides, and creatine. Finally, new knowledge presented herein can be used to guide future research priorities involving meat (including beef) in improving human nutrition, health and well-being.

## Abbreviations

BW: Body weight
CreaT: Creatine transporter
GAT: Gamma-aminobutyrate transporter
PAT: Proton-coupled amino acid transporter
PepT: Peptide transporter
PHT: Peptide/histidine transporter
ROS: Reactive oxygen species
TauT: Taurine transporter

## References

[CR1] Abplanalp W, Haberzettl P, Bhatnagar A (2019). Carnosine supplementation mitigates the deleterious effects of particulate matter exposure in mice. J Am Heart Assoc.

[CR2] Adam M, Spacek P, Hulejova H (1996). Postmenopausal osteoporosis. Treatment with calcitonin and a diet rich in collagen proteins. Cas Lek Cesk.

[CR500] Adhihetty PJ, Beal MF (2008). Creatine and its potential therapeutic value for targeting cellular energy impairment in neurodegenerative diseases. NeuroMol Med.

[CR3] Ahmadi N, Ghanbarinejad V, Ommati MM (2018). Taurine prevents mitochondrial membrane permeabilization and swelling upon interaction with manganese. J Biochem Mol Toxicol.

[CR4] Anderson CMH, Howard A, Walters JRF (2009). Taurine uptake across the human intestinal brush-border membrane is via two transporters: H^+^-coupled PAT1 (SLC36A1) and Na^+^- and Cl^−^-dependent TauT (SLC6A6). J Physiol.

[CR5] Ansurudeen I, Sunkari VG, Grünler J (2012). Carnosine enhances diabetic wound healing in the db/db mouse model of type 2 diabetes. Amino Acids.

[CR6] Ao J, Li B (2012). Amino acid composition and antioxidant activities of hydrolysates and peptide fractions from porcine collagen. Food Sci Technol Int.

[CR7] Artioli GG, Sale C, Jones RL (2019). Carnosine in health and disease. Eur J Sport Sci.

[CR8] Asatoor AM, Bandoh JK, Lant AF (1970). Intestinal absorption of carnosine and its constituent amino acids in man. Gut.

[CR9] Asserin J, Lati E, Shioya T (2015). The effect of oral collagen peptide supplementation on skin moisture and the dermal collagen network. J Cosmetic Dermatol.

[CR10] Avgerinos KI, Spyrou N, Bougioukas KI (2018). Effects of creatine supplementation on cognitive function of healthy individuals. Exp Gerontol.

[CR11] Aydin AF, Kusku-Kiraz Z, Dogru-Abbasoglu S (2010). Effect of carnosine against thioacetamide-induced liver cirrhosis in rat. Peptides.

[CR12] Aydin AF, Kucukgergin C, Ozdemirler-Erata G (2010). The effect of carnosine treatment on prooxidant-antioxidant balance in liver, heart and brain tissues of male aged rats. Biogerontology.

[CR501] Babizhayev MA, Deyev AI (2012). Management of the virulent influenza virus infection by oral formulation of nonhydrolized carnosine and isopeptide of carnosine attenuating proinflammatory cytokine-induced nitric oxide production. Am J Therapeut.

[CR13] Babizhayev MA, Deyev AI, Yermakova VN (2001). N-acetylcarnosine, a natural histidine-containing dipeptide, as a potent ophthalmic drug in treatment of human cataracts. Peptides.

[CR502] Babizhayev MA, Deyev AI, Yegorov YE (2014). L-carnosine modulates respiratory burst and reactive oxygen species production in neutrophil biochemistry and function: may oral dosage form of non-hydrolized dipeptide L-carnosine complement anti-infective anti-influenza flu treatment, prevention and self-care as an alternative to the conventional vaccination?. Curr Clin Pharmacol.

[CR14] Baguet A, Bourgois J, Vanhee L (2010). Important role of muscle carnosine in rowing performance. J Appl Physiol.

[CR15] Baguet A, Everaert I, Achten E (2012). The influence of sex, age and heritability on human skeletal muscle carnosine content. Amino Acids.

[CR503] Balestrino M, Rebaudo R, Lunardi G (1999). Exogenous creatine delays anoxic depolarization and protects from hypoxic damage: dose-effect relationship. Brain Res.

[CR504] Balestrino M, Lensman M, Parodi M (2002). Role of creatine and phosphocreatine in neuronal protection from anoxic and ischemic damage. Amino Acids.

[CR505] Balestrino M, Sarocchi M, Adriano E (2016). Potential of creatine or phosphocreatine supplementation in cerebrovascular disease and in ischemic heart disease. Amino Acids.

[CR16] Baker JS, McCormick MC, Robergs RA (2010). Interaction among skeletal muscle metabolic energy systems during intense exercise. J Nutr Metab.

[CR17] Bala PA, Foster J, Carvelli L (2013). SLC6 transporters: Structure, function, regulation, disease association and therapeutics. Mol Aspects Med.

[CR18] Barbaresi S, Maertens L, Claeys E (2019). Differences in muscle histidine-containing dipeptides in broilers. J Sci Food Agric.

[CR19] Barca A, Ippati S, Urso E (2019). Carnosine modulates the Sp1-Slc31a1/Ctr1 copper-sensing system and influences copper homeostasis in murine CNS-derived cells. Am J Physiol Cell Physiol.

[CR20] Baye E, Ukropec J, de Courten MPJ (2019). Carnosine supplementation reduces plasma soluble transferrin receptor in healthy overweight or obese individuals. Amino Acids.

[CR21] Bellia F, Vecchio G, Rizzarelli E (2014). Carnosinases, their substrates and diseases. Molecules.

[CR22] Bender A, Auer DP, Merl T (2005). Creatine supplementation lowers brain glutamate levels in Huntington's disease. J Neurol.

[CR23] Berezhnoy DS, Stvolinsky SL, Lopachev AV (2019). Carnosine as an effective neuroprotector in brain pathology and potential neuromodulator in normal conditions. Amino Acids.

[CR24] Bertinaria M, Rolando B, Giorgis M (2011). Synthesis, physico-chemical characterization and biological activities of new carnosine derivatives stable in human serum as potential neuroprotective agents. J Med Chem.

[CR25] Blancquaert L, Everaert I, Missinne M (2017). Effects of histidine and β-alanine supplementation on human muscle carnosine storage. Med Sci Sports Exerc.

[CR26] Boldyrev A, Fedorova T, Stepanova M (2008). Carnosine increases efficiency of DOPA therapy of Parkinson’s disease: a pilot study. Rejuvenation Res.

[CR27] Boldyrev AA, Aldini G, Derave W (2013). Physiology and pathophysiology of carnosine. Physiol Rev.

[CR28] Brosnan JT, Brosnan ME (2007). Creatine: endogenous metabolite, dietary, and therapeutic supplement. Annu Rev Nutr.

[CR29] Brüggemann C, Denger K, Cook AM (2004). Enzymes and genes of taurine and isethionate dissimilation in Paracoccus denitrificans. Microbiology.

[CR30] Butterworth CE, Santini R, Perez-Santiago E (1958). The absorption of glycine and its conversion to serine in patients with sprue. J Clin Invest.

[CR31] Calles-Escandon J, Cunningham JJ, Snyder P (1984). Influence of exercise on urea, creatinine, and 3-methylhistidine excretion in normal human subjects. Am J Physiol.

[CR32] Candow DG, Chilibeck PD, Forbes SC (2014). Creatine supplementation and aging musculoskeletal health. Endocrine.

[CR33] Candow DG, Forbes SC, Chilibeck PD (2019). Variables influencing the effectiveness of creatine supplementation as a therapeutic intervention for sarcopenia. Front Nutr.

[CR34] Candow DG, Forbes SC, Chilibeck PD et al (2019) Effectiveness of creatine supplementation on aging muscle and bone: focus on falls prevention and inflammation. J Clin Med 8 10.3390/jcm8040488 PMC6518405 30978926

[CR35] Cararo JH, Streck EL, Schuck PF (2015). Carnosine and related peptides: therapeutic potential in age-related disorders. Aging Dis.

[CR36] Carnegie PR, Hee KP, Bell AW (1982). Ophidine (β-alanyl-l-3-methylhistidine, ‘balenine’) and other histidine dipeptides in pig muscles and tinned hams. J Sci Food Agric.

[CR37] Caruso G, Fresta CG, Martinez-Becerra F (2017). Carnosine modulates nitric oxide in stimulated murine RAW 264.7 macrophages. Mol Cell Biochem.

[CR38] Casey A, Greenhaff PL (2000). Does dietary creatine supplementation play a role in skeletal muscle metabolism and performance?. Am J Clin Nutr.

[CR39] Cella PS, Marinello PC, Borges FH (2019). Creatine supplementation in Walker-256 tumor-bearing rats prevents skeletal muscle atrophy by attenuating systemic inflammation and protein degradation signaling. Eur J Nutr.

[CR40] Chengappa KN, Turkin SR, Desanti S (2012). A preliminary, randomized, double-blind, placebocontrolled trial of L-carnosine to improve cognition in schizophrenia. Schizophr Res.

[CR41] Chez MG, Buchanan CP, Aimonovitch MC (2002). Double-blind, placebo-controlled study of l-carnosine supplementation in children with autistic spectrum disorders. J Child Neurol.

[CR42] Christie DL (2007). Functional insights into the creatine transporter. Subcell Biochem.

[CR43] Cicero AFG, Colletti A (2017). Nutraceuticals and dietary supplements to improve quality of life and outcomes in heart failure patients. Curr Pharm Des.

[CR44] Clifford WM (1922). The effect of cold storage on the carnosine content of muscle. Biochem J.

[CR45] Cook AM, Denger K (2006). Metabolism of taurine in microorganisms: a primer in molecular biodiversity?. Adv Exp Med Biol.

[CR46] Cooper SK, Pandhare J, Donald SP (2008). A novel function for hydroxyproline oxidase in apoptosis through generation of reactive oxygen species. J Biol Chem.

[CR47] Crisafulli DL, Buddhadev HH, Brilla LR (2018). Creatine-electrolyte supplementation improves repeated sprint cycling performance: A double blind randomized control study. J Int Soc Sports Nutr.

[CR48] Culbertson JY, Kreider RB, Greenwood M (2010). Effects of beta-alanine on muscle carnosine and exercise performance: A review of the current literature. Nutrients.

[CR49] Cuzzocrea S, Genovese T, Failla M (2007). Protective effect of orally administered carnosine on bleomycin-induced lung injury. Am J Physiol.

[CR50] da Silva RP, Leonard KA, Jacobs RL (2017). Dietary creatine supplementation lowers hepatic triacylglycerol by increasing lipoprotein secretion in rats fed high-fat diet. J Nutr Biochem.

[CR51] Davey C (1960). The significance of carnosine and anserine in striated skeletal muscle. Arch Biochem Biophysiol.

[CR52] De Benedetto F, Pastorelli R, Ferrario M (2018). Supplementation with Qter^®^ and creatine improves functional performance in COPD patients on long term oxygen therapy. Respir Med.

[CR53] De Carvalho FG, Galan BSM, Santos PC (2017). Taurine: a potential ergogenic aid for preventing muscle damage and protein catabolism and decreasing oxidative stress produced by endurance exercise. Front Physiol.

[CR54] de Courten B, Jakubova M, de Courten MP (2016). Effects of carnosine supplementation on glucose metabolism: pilot clinical trial. Obesity.

[CR55] Deal CL, Moskowitz RW (1999). Nutraceuticals as therapeutic agents in osteoarthritis: the role of glucosamine, chondroitin sulfate, and collagen hydrolysate. Rheum Dis Clin N Am.

[CR56] Deldicque L, Decombaz J, Zbinden Foncea H (2008). Kinetics of creatine ingested as a food ingredient. Eur J Appl Physiol.

[CR57] Devlin TM (1992). Textbook of biochemistry with clinical correlations.

[CR58] Derave W, Marescau B, Vanden Eede E (2004). Plasma guanidino compounds are altered by oral creatine supplementation in healthy humans. J Appl Physiol.

[CR59] Derave W, Ozdemir MS, Harris RC (2007). Beta-alanine supplementation augments muscle carnosine content and attenuates fatigue during repeated isokinetic contraction bouts in trained sprinters. J Appl Physiol.

[CR60] Derave W, De Courten B, Baba SP (2019). An update on carnosine and anserine research. Amino Acids.

[CR61] Ding Q, Tanigawa K, Kaneko J (2018). Anserine/carnosine supplementation preserves blood flow in the prefrontal brain of elderly people carrying APOE e4. Aging Dis.

[CR62] Dolan E, Gualano B, Rawson ES (2019). Beyond muscle: the effects of creatine supplementation on brain creatine, cognitive processing and traumatic brain injury. Eur J Sport Sci.

[CR63] Drozak J, Veiga-da-Cunha M, Vertommen D (2010). Molecular identification of carnosine synthase as ATP-grasp domain-containing protein 1 (ATPGD1). J Biol Chem.

[CR64] Dursun N, Taskin E, Ozturk F (2011). Protection against adriamycin-induced cardiomyopathy by carnosine in rats: role of endogenous antioxidants. Biol Trace Elem Res.

[CR65] Eichhorn E, van der Ploeg JR, Kertesz MA (1997). Characterization of alpha-ketoglutarate-dependent taurine dioxygenase from *Escherichia coli*. J Biol Chem.

[CR66] El Idrissi A (2019). Taurine regulation of neuroendocrine function. Adv Exp Med Biol.

[CR67] Elbarbary NS, Ismail EAR, El-Naggar AR (2017). The effect of 12 weeks carnosine supplementation on renal functional integrity and oxidative stress in pediatric patients with diabetic nephropathy: a randomized placebo-controlled trial. Pediatr Diabetes.

[CR68] Everaert I, Mooyaart A, Baguet A (2011). Vegetarianism, female gender and increasing age, but not CNDP1 genotype, are associated with reduced muscle carnosine levels in humans. Amino Acids.

[CR69] Everaert I, De Naeyer H, Taes Y (2013). Gene expression of carnosine-related enzymes and transporters in skeletal muscle. Eur J Appl Physiol.

[CR70] Everaert I, Baron G, Barbaresi S (2019). Development and validation of a sensitive LC–MS/MS assay for the quantification of anserine in human plasma and urine and its application to pharmacokinetic study. Amino Acids.

[CR71] Food and Drug Administration (FDA), Center for Drug Evaluation and Research (CDER), U.S. Department of Health and Human Services (2005). Guidance for industry: estimating the maximum safe starting dose in initial clinical trials for therapeutics in adult healthy volunteers.

[CR72] Fairman CM, Kendall KL, Hart NH (2019). The potential therapeutic effects of creatine supplementation on body composition and muscle function in cancer. Crit Rev Oncol Hematol.

[CR73] Fedorova TN, Devyatov AA, Berezhnoi DS (2018). Oxidative status in different areas of the cerebral cortex of Wistar rats during focal ischemia and its modulation with carnosine. Bull Exp Biol Med.

[CR74] Fernley HN (1971). Mammalian alkaline phosphatases Enzymes.

[CR75] Foley MH, O’Flaherty S, Barrangou R (2019). Bile salt hydrolases: gatekeepers of bile acid metabolism and host-microbiome crosstalk in the gastrointestinal tract. PLoS Pathog.

[CR76] Fortalezas S, Marques-da-Silva D, Gutierrez-Merino C (2018). Creatine protects against cytosolic calcium dysregulation, mitochondrial depolarization and increase of reactive oxygen species production in rotenone-induced cell death of cerebellar granule neurons. Neurotox Res.

[CR77] Fouad AA, Morsy MA, Gomaa W (2008). Protective effect of carnosine against cisplatin-induced nephrotoxicity in mice. Environ Toxicol Pharmacol.

[CR78] Gardner ML, Illingworth KM, Kelleher J (1991). Intestinal absorption of the intact peptide carnosine in man, and comparison with intestinal permeability to lactulose. J Physiol.

[CR79] Geggel H, Ament M, Heckenlively J (1985). Nutritional requirement for taurine in patients receiving long-term, parenteral nutrition. N Engl J Med.

[CR506] Genc G, Okuyucu A, Meydan BC (2014). Effect of free creatine therapy on cisplatin-induced renal damage. Ren Fail.

[CR80] Gibson NR, Jahoor F, Ware L (2002). Endogenous glycine and tyrosine production is maintained in adults consuming a marginal-protein diet. Am J Clin Nutr.

[CR508] Gottardi W, Nagl M (2010). N-chlorotaurine, a natural antiseptic with outstanding tolerability. J Antimicrob Chemother.

[CR81] Gray GE, Landel AM, Meguid MM (1994). Taurine-supplemented total parenteral nutrition and taurine status of malnourished cancer patients. Nutrition.

[CR82] Gray SR, Soderlund K, Watson M (2011). Skeletal muscle ATP turnover and single fibre ATP and PCr content during intense exercise at different muscle temperatures in humans. Pflügers Archiv.

[CR83] Grillenberger M, Neumann CG, Murphy SP (2003). Food supplements have a positive impact on weight gain and the addition of animal source foods increases lean body mass of Kenyan school children. J Nutr.

[CR84] Harding JW, O'Fallon JV (1979). The subcellular distribution of carnosine, carnosine synthetase, and carnosinase in mouse olfactory tissues. Brain Res.

[CR85] Harris CI, Milne G (1986). The identification of the N tau-methyl histidine-containing dipeptide, balenine, in muscle extracts from various mammals and the chicken. Comp Biochem Physiol B.

[CR86] Harris RC, Tallon MJ, Dunnett M (2006). The absorption of orally supplied beta-alanine and its effect on muscle carnosine synthesis in human vastus lateralis. Amino Acids.

[CR87] Harris RC, Wise JA, Price KA (2012). Determinants of muscle carnosine content. Amino Acids.

[CR88] Hayes KC, Carey RE, Schmidt SY (1975). Retinal degeneration associated with taurine deficiency in the cat. Science.

[CR89] Healy MA, Thirumurthi S, You YN (2019). Screening high-risk populations for colon and rectal cancers. J Surg Oncol.

[CR90] Heimesaat MM, Heilmann K, Kühl AA (2012). The synthetic hydroxyproline-containing collagen analogue (Gly-Pro-Hyp)_10_ ameliorates acute DSS colitis. Eur J Microbiol Immunol.

[CR91] Hickner RC, Dyck DJ, Sklar J (2010). Effect of 28 days of creatine ingestion on muscle metabolism and performance of a simulated cycling road race. J Int Soc Sports Nutr.

[CR92] Hill CA, Harris RC, Kim HJ (2007). Influence of beta-alanine supplementation on skeletal muscle carnosine concentrations and high intensity cycling capacity. Amino Acids.

[CR93] Hipkiss AR, Brownson C (2000). A possible new role for the anti-ageing peptide carnosine. Cell Mol Life Sci.

[CR94] Hipkiss AR, Gaunitz F (2014). Inhibition of tumour cell growth by carnosine: some possible mechanisms. Amino Acids.

[CR95] Hirohiko M, Kazushige G, Toshitsugu Y (2006). Efects of carnosine and anserine supplementation on relatively high intensity endurance. Int J Sport Health Sci.

[CR96] Hisatsune T, Kaneko J, Kurashige H (2016). Effect of anserine/carnosine supplementation on verbal episodic memory in elderly people. J Alzheimers Dis.

[CR97] Hofmann AF (1999). The continuing importance of bile acids in liver and intestinal disease. Arch Intern Med.

[CR98] Holt LE, Albanese AA (1944). Observations on amino acid deficiencies in man. Trans Assoc Am Physicians.

[CR99] Horinishi H, Grillo M, Margolis FL (1978). Purification and characterization of carnosine synthetase from mouse olfactory bulbs. J Neurochem.

[CR100] Horning MS, Blakemore LJ, Trombley PQ (2000). Endogenous mechanisms of neuroprotection: role of zinc, copper, and carnosine. Brain Res.

[CR509] Hou YQ, Wu G (2017). Nutritionally nonessential amino acids: a misnomer in nutritional sciences. Adv Nutr.

[CR510] Hou YQ, Wu G (2018). Nutritionally essential amino acids. Adv Nutr.

[CR101] Hou YQ, Yin YL, Wu G (2015). Dietary essentiality of "nutritionally nonessential amino acids" for animals and humans. Exp Biol Med.

[CR102] Hou YQ, He WL, Hu SD (2019). Composition of polyamines and amino acids in plant-source foods for human consumption. Amino Acids.

[CR103] Houjeghani S, Kheirouri S, Faraji E (2018). L-Carnosine supplementation attenuated fasting glucose, triglycerides, advanced glycation end products, and tumor necrosis factor-α levels in patients with type 2 diabetes: a double-blind placebo-controlled randomized clinical trial. Nutr Res.

[CR104] Hsieh SL, Hsieh S, Lai PY (2019). Carnosine suppresses human colorectal cell migration and intravasation by regulating EMT and MMP expression. Am J Chin Med.

[CR105] Hu S, Nawaratna G, Long BD (2017). The hydroxyproline–glycine pathway for glycine synthesis in neonatal pigs. J Anim Sci.

[CR106] Hultman E, Söderlund K, Timmons JA (1996). Muscle creatine loading in men. J Appl Physiol.

[CR107] Hummer E, Suprak DN, Buddhadev HH (2019). Creatine electrolyte supplement improves anaerobic power and strength: a randomized double-blind control study. J Int Soc Sports Nutr.

[CR108] Huxtable RJ (1992). Physiological actions of taurine. Physiol Rev.

[CR109] Inoue N, Sugihara F, Wang X (2016). Ingestion of bioactive collagen hydrolysates enhance facial skin moisture and elasticity and reduce facial ageing signs in a randomised double-blind placebo-controlled clinical study. J Sci Food Agric.

[CR110] Institute of Medicine (IOM, 2006). Protein and amino acids. Dietary reference intakes: the essential guide to nutrient requirements. Institute of Medicine, National Academies Press, Washington

[CR111] Iovine B, Guardia F, Irace C (2016). L-carnosine dipeptide overcomes acquired resistance to 5-fluorouracil in HT29 human colon cancer cells via downregulation of HIF1-alpha and induction of apoptosis. Biochimie.

[CR112] Ito T, Schaffer S, Azuma J (2014). The effect of taurine on chronic heart failure: actions of taurine against catecholamine and angiotensin II. Amino Acids.

[CR113] Jacobsen JG, Smith LH (1968). Biochemistry and physiology of taurine and taurine derivatives. Physiol Rev.

[CR114] Jäger R, Harris RC, Purpura M, Francaux M (2007). Comparison of new forms of creatine in raising plasma creatine levels. J Int Soc Sports Nutr.

[CR115] Jäger R, Purpura M, Shao A (2011). Analysis of the efficacy, safety, and regulatory status of novel forms of creatine. Amino Acids.

[CR116] Jamshidzadeh A, Heidari R, Abasvali M (2017). Taurine treatment preserves brain and liver mitochondrial function in a rat model of fulminant hepatic failure and hyperammonemia. Biomed Pharmacother.

[CR117] Jamshidzadeh A, Heidari R, Latifpour Z (2017). Carnosine ameliorates liver fibrosis and hyperammonemia in cirrhotic rats. Clin Res Hepatol Gastroenterol.

[CR118] Johnson P, Hammer JL (1992). Histidine dipeptide levels in ageing and hypertensive rat skeletal and cardiac muscles. Comp Biochem Physiol B.

[CR119] Johnson P, Fedyna JS, Schindzielorz A (1982). Regulation of muscle phosphorylase activity by carnosine and anserine. Biochem Biophys Res Commun.

[CR120] Johnston BC, Zeraatkar D, Han MA (2019). Unprocessed red meat and processed meat consumption: dietary guideline recommendations from the NutriRECS consortium. Ann Intern Med.

[CR121] Ji Y, Dai ZL, Sun SQ (2018). Hydroxyproline attenuates dextran sulfate sodium-induced colitis in mice: involvement of the NF-κB signaling and oxidative stress. Mol Nutr Food Res.

[CR122] Juhasz I, Kopkane JP, Hajdu P (2018). Creatine supplementation supports the rehabilitation of adolescent fin swimmers in tendon overuse injury cases. J Sports Sci Med.

[CR123] Jong CJ, Azuma J, Schaffer S (2012). Mechanisms underlying the antioxidant activity of taurine: prevention of mitochondrial oxidant production. Amino Acids.

[CR124] Kaneko J, Enya A, Enomoto K (2017). Anserine (beta-alanyl-3-methyl-l-histidine) improves neurovascular-unit dysfunction and spatial memory in aged AβPPswe/PSEN1dE9 Alzheimer's-model mice. Sci Rep.

[CR125] Katakura Y, Totsuka M, Imabayashi E et al. (2017) Anserine/carnosine supplementation suppresses the expression of the inflammatory chemokine CCL24 in peripheral blood mononuclear cells from elderly people. Nutrients 9(11) 10.3390/nu9111199 PMC5707671 29088099

[CR126] Kausar T, Hanan E, Ayob O (2019). A review on functional ingredients in red meat products. Bioinformation.

[CR127] Kawahara M, Tanaka KI, Kato-Negishi M (2018). Zinc, carnosine, and neurodegenerative diseases. Nutrients.

[CR128] Keller TC, Gordon PV (1991). Discrete subcellular localization of a cytoplasmic and a mitochondrial isozyme of creatine kinase in intestinal epithelial cells. Cell Motil Cytoskelet.

[CR129] Kenéz A, Warnken T, Feige K (2018). Lower plasma trans-4-hydroxyproline and methionine sulfoxide levels are associated with insulin dysregulation in horses. BMC Vet Res.

[CR130] Knight J, Jiang J, Assimos DG (2006). Hydroxyproline ingestion and urinary oxalate and glycolate excretion. Kidney Int.

[CR131] Kohen R, Yamamoto Y, Cundy KC (1988). Antioxidant activity of carnosine, homocarnosine, and anserine present in muscle and brain. Proc Natl Acad Sci USA.

[CR511] Komi PV, Karlsson J (1978). Skeletal muscle fibre types, enzyme activities and physical performance in young males and females. Acta Physiol Scand.

[CR132] König D, Oesser S, Scharla S (2018). Specific collagen peptides improve bone mineral density and bone markers in postmenopausal women. Nutrients.

[CR133] Kreider RB, Kalman DS, Antonio J (2017). International Society of Sports Nutrition position stand: safety and efficacy of creatine supplementation in exercise, sport, and medicine. J Int Soc Sports Nutr.

[CR134] Kristensen CA, Askenasy N, Jain RK (1999). Creatine and cyclocreatine treatment of human colon adenocarcinoma xenografts: ^31^P and ^1^H magnetic resonance spectroscopic studies. Br J Cancer.

[CR135] Kubomura D, Matahira Y, Masui A (2009). Intestinal absorption and blood clearance of l-histidine-related compounds after ingestion of anserine in humans and comparison to anserinecontaining diets. J Agric Food Chem.

[CR136] Kubomura D, Matahira Y, Nagai K (2010). Effect of anserine ingestion on hyperglycemia and the autonomic nerves in rats and humans. Nutr Neurosci.

[CR137] Kume S, Yamato M, Tamura Y (2015). Potential biomarkers of fatigue identified by plasma metabolome analysis in rats. PLoS ONE.

[CR138] Kusubata M, Koyama Y, Tometsuka C (2015). Detection of endogenous and food-derived collagen dipeptide prolylhydroxyproline (Pro-Hyp) in allergic contact dermatitis-affected mouse ear. Biosci Biotechnol Biochem.

[CR139] Laidlaw SA, Shultz TD, Cecchino JT (1988). Plasma and urine taurine levels in vegans. Am J Clin Nutr.

[CR140] Lambert IH, Hansen DB (2011). Regulation of taurine transport systems by protein kinase CK2 in mammalian cells. Cell Physiol Biochem.

[CR141] Lawler JM, Barnes WS, Wu G (2002). Direct antioxidant properties of creatine. Biochem Biophys Res Commun.

[CR142] Lee JW, Miyawaki H, Bobst EV (1999). Improved functional recovery of ischemic rat hearts due to singlet oxygen scavengers histidine and carnosine. J Mol Cell Cardiol.

[CR143] Lee YT, Hsu CC, Lin MH (2005). Histidine and carnosine delay diabetic deterioration in mice and protect human low density lipoprotein against oxidation and glycation. Eur J Pharmacol.

[CR144] Lenney JF, Peppers SC, Kucera-Orallo CM (1985). Characterization of human tissue carnosinase. Biochem J.

[CR145] Lensman M, Korzhevskii DE, Mourovets VO (2006). Intracerebroventricular administration of creatine protects against damage by global cerebral ischemia in rat. Brain Res.

[CR146] Leroy F, Cofnas N (2019). Should dietary guidelines recommend low red meat intake?. Crit Rev Food Sci Nutr.

[CR147] Lexell J, Taylor CC, Sjostrom M (1988). What is the cause of ageing atrophy? Total number, size, and proportion of different fiber types studied in whole vastus lateralis muscle from 15- to 83-year-old men. J Neurol Sci.

[CR148] Li P, Wu G (2018). Roles of dietary glycine, proline and hydroxyproline in collagen synthesis and animal growth. Amino Acids.

[CR149] Li C, Cao L, Zeng Q (2005). Taurine may prevent diabetic rats from developing cardiomyopathy also by downregulating angiotensin II type2 receptor expression. Cardiovasc Drugs Ther.

[CR150] Li YF, He RR, Tsoi B (2012). Bioactivities of chicken essence. J Food Sci.

[CR151] Lillie JW, O’Keefe M, Valinski H (1993). Cyclocreatine (1-carboxymethyl-2-iminoimidazolidine) inhibits growth of a broad spectrum of cancer cells derived from solid tumours. Cancer Res.

[CR152] Liu WH, Liu TC, Yin MC (2008). Beneficial effects of histidine and carnosine on ethanol-induced chronic liver injury. Food Chem Toxicol.

[CR153] Liu Y, Cotillard A, Vatier C (2015). A dietary supplement containing cinnamon, chromium and carnosine decreases fasting plasma glucose and increases lean mass in overweight or obese pre-diabetic subjects: a randomized, placebo-controlled trial. PLoS ONE.

[CR154] Lombardi C, Carubelli V, Lazzarini V (2015). Effects of oral administration oforodispersible levo-carnosine on quality of life and exercise performance in patientswith chronic heart failure. Nutrition.

[CR155] Lowry M, Hall DE, Brosnan JT (1985). Hydroxyproline metabolism by the rat kidney: distribution of renal enzymes of hydroxyproline catabolism and renal conversion of hydroxyproline to glycine and serine. Metabolism.

[CR156] Lupi A, Tenni R, Rossi A (2008). Human prolidase and prolidase deficiency: an overview on the characterization of the enzyme involved in proline recycling and on the effects of its mutations. Amino Acids.

[CR157] Ma XY, Jiang ZY, Lin YC (2010). Dietary supplementation with carnosine improves antioxidant capacity and meat quality of finishing pigs. J Anim Physiol Anim Nutr.

[CR158] Mannion AF, Jakeman PM, Dunnett M (1992). Carnosine and anserine concentrations in the quadriceps femoris muscle of healthy humans. Eur J Appl Physiol Occup Physiol.

[CR159] Masuoka N, Yoshimine C, Hori M et al (2019) Effects of anserine/carnosine supplementation on mild cognitive impairment with APOE4. Nutrients 11 10.3390/nu11071626 PMC6683059 31319510

[CR160] Mateescu RG, Garmyn AJ, O’Neil MA (2012). Genetic parameters for carnitine, creatine, creatinine, carnosine, and anserine concentration in longissimus muscle and their association with palatability traits in Angus cattle. J Anim Sci.

[CR161] Matsumoto H, Ohara H, Itoh K (2006). Clinical effect of fish type I collagen hydrolysate on skin properties. ITE Lett.

[CR162] Matsumura Y, Kita S, Ono H (2002). Preventive effect of a chicken extract on the development of hypertension in stroke-prone spontaneously hypertensive rats. Biosci Biotechnol Biochem.

[CR163] Matthews JJ, Artioli GG, Turner MD (2019). The physiological roles of carnosine and β-aalanine in exercising human skeletal muscle. Med Sci Sports Exerc.

[CR164] McCarty MF, O’Keefe JH, DiNicolantonio JJ (2018). Dietary glycine is rate-limiting for glutathione synthesis and may have broad potential for health protection. Ochsner J.

[CR165] McGilvery RW, Murray TW (1974). Calculated equilibria of phosphocreatine and adenosine phosphates during utilization of high energy phosphate by muscle. J Biol Chem.

[CR166] Meléndez-Hevia E, De Paz-Lugo P, Cornish-Bowden A (2009). A weak link in metabolism: the metabolic capacity for glycine biosynthesis does not satisfy the need for collagen synthesis. J Biosci.

[CR167] Milić S, Bogdanović Pristov J, Mutavdžić D (2015). The relationship of physicochemical properties to the antioxidative activity of free amino acids in Fenton system. Environ Sci Technol.

[CR168] Militante JD, Lombardini JB (2002). Treatment of hypertension with oral taurine: experimental and clinical studies. Amino Acids.

[CR169] Militante JD, Lombardini JB, Schaffer SW (2000). The role of taurine in the pathogenesis of the cardiomyopathy of insulin-dependent diabetes mellitus. Cardiovasc Res.

[CR170] Miller EE, Evans AE, Cohn M (1993). Inhibition of rate of tumour growth by creatine and cyclocreatine. Proc Natl Acad Sci USA.

[CR171] Moskowitz RW (2000). Role of collagen hydrolysate in bone and joint disease. Semin Arthritis Rheum.

[CR172] Murphy R, McConell G, Cameron-Smith D (2001). Creatine transporter protein content, localization, and gene expression in rat skeletal muscle. Am J Physiol.

[CR173] Myllyharju J, Koivunen P (2013). Hypoxia-inducible factor prolyl 4-hydroxylases: common and specific roles. Biol Chem.

[CR174] Nagai K, Misonou Y, Fujisaki Y (2019). Topical application of l-carnosine to skeletal muscle excites the sympathetic nerve innervating the contralateral skeletal muscle in rats. Amino Acids.

[CR175] Nakatani S, Mano H, Sampei C (2009). Chondroprotective effect of the bioactive peptide prolyl-hydroxyproline in mouse articular cartilage in vitro and in vivo. Osteoarthr Cartil.

[CR176] Nakatsuru Y, Murase-Mishiba Y, Bessho-Tachibana M (2018). Taurine improves glucose tolerance in STZ-induced insulin-deficient diabetic mice. Diabetol Int.

[CR177] Nelson ME, Hamm MW, Hu FB (2016). Alignment of healthy dietary patterns and environmental sustainability: a systematic review. Adv Nutr.

[CR178] Nelson MM, Builta ZJ, Monroe TB (2019). Biochemical characterization of the catecholaldehyde reactivity of l-carnosine and its therapeutic potential in human myocardium. Amino Acids.

[CR179] Neumann CG, Murphy SP, Gewa C (2007). Meat supplementation improves growth, cognitive, and behavioral outcomes in Kenyan children. J Nutr.

[CR180] Ng RH, Marshall FD (1978). Regional and subcellular distribution of homocarnosine-carnosine synthetase in the central nervous system of rats. J Neurochem.

[CR181] Offengenden M, Chakrabarti S, Wu J (2018). Chicken collagen hydrolysates differentially mediate anti-inflammatory activity and type I collagen synthesis on human dermal fibroblasts. Food Sci Hum Wellness.

[CR182] Ohara H, Matsumoto H, Itoh K (2007). Comparison of quantity and structures of hydroxyproline-containing peptides in human blood after oral ingestion of gelatin hydrolysates from different sources. J Agric Food Chem.

[CR183] Ohsawa Y, Hagiwara H, Nishimatsu SI (2019). Taurine supplementation for prevention of stroke-like episodes in MELAS: a multicentre, open-label, 52-week phase III trial. J Neurol Neurosurg Psychiatry.

[CR184] Oppermann H, Alvanos A, Seidel C (2019). Carnosine influences transcription via epigenetic regulation as demonstrated by enhanced histone acetylation of the pyruvate dehydrogenase kinase 4 promoter in glioblastoma cells. Amino Acids.

[CR185] Osawa Y, Mizushige T, Jinno S (2018). Absorption and metabolism of orally administered collagen hydrolysates evaluated by the vascularly perfused rat intestine and liver in situ. Biomed Res (Tokyo).

[CR512] Osbakken M, Ito K, Zhang D (1992). Creatine and cyclocreatine effects on ischemic myocardium: 31P nuclear magnetic resonance evaluation of intact heart. Cardiology.

[CR186] Page LK, Jeffries O, Waldron M (2019). Acute taurine supplementation enhances thermoregulation and endurance cycling performance in the heat. Eur J Sport Sci.

[CR187] Pal A, Roy A, Ray M (2016). Creatine supplementation with methylglyoxal: a potent therapy for cancer in experimental models. Amino Acids.

[CR188] Park YJ, Volpe SL, Decker EA (2005). Quantitation of carnosine in human plasma after dietary consumption of beef. J Agric Food Chem.

[CR189] Paulucio D, Costa BM, Santos CGM (2017). Taurine supplementation improves economy of movement in the cycle test independently of the detrimental effects of ethanol. Biol Sport.

[CR190] Pavlov AR, Revina AA, Dupin AM (1993). The mechanism of interaction of carnosine with superoxide radicals in water solutions. Biochim Biophys Acta.

[CR191] Peeters BM, Lantz CD, Mayhew JL (1999). Effect of oral creatine monohydrate and creatine phosphate supplementation on maximal strength indices, body composition, and blood pressure. J Strength Cond Res.

[CR192] Peng HC, Lin SH (2004). Effects of chicken extract on antioxidative status and liver protection under oxidative stress. J Nutr Sci Vitaminol (Tokyo).

[CR513] Perasso L, Spallarossa P, Gandolfo C (2013). Therapeutic use of creatine in brain or heart ischemia: available data and future perspectives. Med Res Rev.

[CR514] Persky AM, Brazeau GA (2001). Clinical pharmacology of the dietary supplement creatine monohydrate. Pharmacol Rev.

[CR193] Peters V, Calabrese V, Forsberg E et al. (2018) Protective actions of anserine under diabetic conditions. Int J Mol Sci 19 10.3390/ijms19092751 PMC6164239 30217069

[CR194] Pfister F, Riedl E, Wang Q (2011). Oral carnosine supplementation prevents vascular damage in experimental diabetic retinopathy. Cell Physiol Biochem.

[CR195] Phang JM, Liu W, Zabirnyk O (2010). Proline metabolism and microenvironmental stress. Annu Rev Nutr.

[CR196] Prass K, Royl G, Lindauer U (2007). Improved reperfusion and neuroprotection by creatine in a mouse model of stroke. J Cereb Blood Flow Metab.

[CR197] Proksch E, Segger D, Degwert J (2014). Oral supplementation of specific collagen peptides has beneficial effects on human skin physiology. Skin Pharmacol Physiol.

[CR515] Rahimi R (2011). Creatine supplementation decreases oxidative DNA damage and lipid peroxidation induced by a single bout of resistance exercise. J Strength Cond Res.

[CR198] Ra SG, Choi Y, Akazawa N (2019). Effects of taurine supplementation on vascular endothelial function at rest and after resistance exercise. Adv Exp Med Biol.

[CR199] Read WO, Welty JD (1962). Synthesis of taurine and isethionic acid by dog heart slices. J Biol Chem.

[CR516] Ren WK, Yin YL, Zhou BY, Calder P, Kulkarni AD (2018). Roles of arginine in cell-mediated and humoral immunity. Nutrition, Immunity, and Infection.

[CR200] Ririe DG, Roberts PR, Shouse MN (2000). Vasodilatory actions of the dietary peptide carnosine. Nutrition.

[CR201] Rodriguez MC, MacDonald JR, Mahoney DJ (2007). Beneficial effects of creatine, CoQ10, and lipoic acid in mitochondrial disorders. Muscle Nerve.

[CR202] Rogerson D (2017). Vegan diets: practical advice for athletes and exercisers. J Int Soc Sports Nutr.

[CR203] Rokicki J, Li L, Imabayashi E (2015). Daily carnosine and anserine supplementation alters verbal episodic memory and resting state network connectivity in healthy elderly adults. Front Aging Neurosci.

[CR204] Roos MR, Rice CL, Vandervoort AA (1997). Age-related changes in motor unit function. Muscle Nerve.

[CR205] Rose WC (1957). The amino acid requirements of adult man. Nutr Abstr Rev Ser Hum Exp.

[CR206] Sadikali F, Darwish R, Watson WC (1975). Carnosinase activity of human gastrointestinal mucosa. Gut.

[CR207] Sak D, Erdenen F, Müderrisoglu C (2019). The Relationship between plasma taurine levels and diabetic complications in patients with type 2 diabetes mellitus. Biomolecules.

[CR208] Sakae K, Yanagisawa H (2014). Oral treatment of pressure ulcers with polaprezinc (zinc L-carnosine complex): 8-week open-label trial. Biol Trace Elem Res.

[CR209] Sale C, Saunders B, Harris RC (2010). Effect of beta-alanine supplementation on muscle carnosine concentrations and exercise performance. Amino Acids.

[CR210] Salomons GS, van Dooren SJM, Verhoeven NM (2001). X-linked creatine-transporter gene (SLC6A8) defect: A new creatine deficiency syndrome. Am J Hum Genet.

[CR211] Santacruz L, Jacobs DO (2016). Structural correlates of the creatine transporter function regulation: the undiscovered country. Amino Acids.

[CR212] Santos-Silva JC, Ribeiro RA, Vettorazzi JF (2015). Taurine supplementation ameliorates glucose homeostasis, prevents insulin and glucagon hypersecretion, and controls β, α, and δ-cell masses in genetic obese mice. Amino Acids.

[CR213] Sarkar P, Basak P, Ghosh S (2017). Prophylactic role of taurine and its derivatives against diabetes mellitus and its related complications. Food Chem Toxicol.

[CR214] Sato K, Jimi S, Kusubata M (2019). Generation of bioactive prolyl-hydroxyproline (Pro-Hyp) by oral administration of collagen hydrolysate and degradation of endogenous collagen. Int J Food Sci Technol.

[CR215] Schaffer S, Kim HW (2018). Effects and mechanisms of taurine as a therapeutic agent. Biomol Ther (Seoul).

[CR216] Schaffer SW, Azuma J, Mozaffari M (2009). Role of antioxidant activity of taurine in diabetes. Can J Physiol Pharmacol.

[CR517] Scheer M, Bischoff AM, Kruzliak P (2016). Creatine and creatine pyruvate reduce hypoxia-induced effects on phrenic nerve activity in the juvenile mouse respiratory system. Exp Mol Pathol.

[CR217] Schön M, Mousa A, Berk M (2019). The potential of carnosine in brain-related disorders: a comprehensive review of current evidence. Nutrients.

[CR218] Seidel U, Huebbe P, Rimbach G (2019). Taurine: a regulator of cellular redox-homeostasis and skeletal muscle function. Mol Nutr Food Res.

[CR219] Sewell DA, Harris RC, Marlin DJ (1992). Estimation of the carnosine content of different fibre types in the middle gluteal muscle of the thoroughbred horse. J Physiol.

[CR220] Shao L, Li QH, Tan Z (2004). l-carnosine reduces telomere damage and shortening rate in cultured normal fibroblasts. Biochem Biophys Res Commun.

[CR221] Shen H, Goldberg MP (2012). Creatine pretreatment protects cortical axons from energy depletion in vitro. Neurobiol Dis.

[CR222] Shigemura Y, Iwai K, Morimatsu F (2009). Effect of prolyl-hydroxyproline (Pro-Hyp), a food-derived collagen peptide in human blood, on growth of fibroblasts from mouse skin. J Agric Food Chem.

[CR223] Shigemura Y, Akaba S, Kawashima E (2011). Identification of a novel food-derived collagen peptide, hydroxyprolyl-glycine, in human peripheral blood by pre-column derivatisation with phenyl isothiocyanate. Food Chem.

[CR224] Shigemura Y, Kubomura D, Sato Y (2014). Dose-dependentchanges in the levels of free and peptide forms of hydroxyproline in human plasma after collagen hydrolysateingestion. Food Chem.

[CR225] Shimada K, Jong CJ, Takahashi K (2015). Role of ROS production and turnover in the antioxidant activity of taurine. Adv Exp Med Biol.

[CR226] Sirdah MM (2015). Protective and therapeutic effectiveness of taurine in diabetes mellitus: a rationale for antioxidant supplementation. Diabetes Metab Syndr.

[CR227] Sjostrom H, Noren O, Josefsson L (1973). Purification and specificity of pig intestinal prolidase. Biochim Biophys Acta.

[CR228] Smith RN, Agharkar AS, Gonzales EB (2014) A review of creatine supplementation in age-related diseases: more than a supplement for athletes. F1000Research 3:222 10.12688/f1000research.5218.1 PMC4304302 25664170

[CR229] Spelnikov D, Harris RC (2019). A kinetic model of carnosine synthesis in human skeletal muscle. Amino Acids.

[CR230] Starck CS, Wolfe RR, Moughan PJ (2018). Endogenous amino acid losses from the gastrointestinal tract of the adult human—a auantitative model. J Nutr.

[CR231] Sturman JA, Hayes KC (1980). The biology of taurine in nutrition and development. Adv Nutr Res.

[CR232] Sturman JA (1993). Taurine in development. Physiol Rev.

[CR233] Suzuki Y, Ito O, Takahashi H (2004). The effect of sprint training on skeletal muscle carnosine in humans. Int J Sport Health Sci.

[CR234] Szterk A, Roszko M (2014). Simultaneous determination of free amino acids, L-carnosinem purine, pyrimidine, and nucleosides in meat by liquid chromatography/single quadrupole mass spectrometry. J Liquid Chromatogr Relat Technol.

[CR235] Szcześniak D, Budzeń S, Kopeć W (2014). Anserine and carnosine supplementation in the elderly: effects on cognitive functioning and physical capacity. Arch Gerontol Geriatr.

[CR236] Tallon MJ, Harris RC, Boobis LH (2005). The carnosine content of vastus lateralis is elevated in resistancetrained bodybuilders. J Strength Cond Res.

[CR237] Tanaka M, Koyama Y, Nomura Y (2009). Effects of collagen peptide ingestion on UV-B-induced skin damage. Biosci Biotechnol Biochem.

[CR238] Tanida M, Shen J, Kubomura D (2010). Effects of anserine on the renalsympathetic nerve activity and blood pressure in urethane-anesthetized rats. Physiol Res.

[CR239] Tanokura M, Tasumi M, Miyazawa T (1976). ^1^H nuclear magnetic resonance studies of histidine containing di and tripeptides. Estimation of the effects of charged groups on the p*K*a value of the imidiazole ring. Biopolymers.

[CR240] Thornton KJ, Richard RP, Colle MJ (2015). Effects of dietary potato by-product and rumen-protected histidine on growth, carcass characteristics and quality attributes of beef. Meat Sci.

[CR241] Trask RV, Billadello JJ (1990). Tissue-specific distribution and developmental regulation of M and B creatine kinase mRNAs. Biochim Biophys Acta.

[CR242] Tsuruoka N, Yamato R, Sakai Y (2007). Promotion by collagen tripeptide of type I collagen gene expression in human osteoblastic cells and fracture healing of rat femur. Biosci Biotechnol Biochem.

[CR243] USDA (2018) Economic research service. Livestock, dairy, and poultry outlook. https://www.ers.usda.gov/. Accessed 16 Aug 2019

[CR244] Uzhova I, Peñalvo JL (2019). Mediterranean diet and cardio-metabolic health: what is the role of meat?. Eur J Clin Nutr.

[CR245] Valman HB, Brown RJK, Palmer T (1971). Protein intake and plasma amino acids of infants of low birth weight. Br Med J.

[CR518] Vatansever F, de Melo WCMA, Avci P (2013). Antimicrobial strategies centered around reactive oxygen species - bactericidal antibiotics, photodynamic therapy and beyond. FEMS Microbiol Rev.

[CR246] Vidot H, Cvejic E, Carey S (2018). Randomised clinical trial: oral taurine supplementation versus placebo reduces muscle cramps in patients with chronic liver disease. Aliment Pharmacol Ther.

[CR247] Waldron M, Patterson SD, Tallent J (2018). The effects of an oral taurine dose and supplementation period on endurance exercise performance in humans: a meta-analysis. Sports Med.

[CR248] Waldron M, Patterson SD, Jeffries O (2019). Oral taurine improves critical power and severe-intensity exercise tolerance. Amino Acids.

[CR249] Wang Z, Shen W, Kotler DP (2003). Total body protein: A new cellular level mass and distribution prediction model. Am J Clin Nutr.

[CR250] Wang CC, Fang CC, Lee YH et al. (2018) Effects of 4-week creatine supplementation combined with complex training on muscle damage and sport performance. Nutrients 10 10.3390/nu10111640 PMC6265971 30400221

[CR251] Watanabe-Kamiyama M, Shimizu M, Kamiyama S (2010). Absorption and effectiveness of orally administered low molecular weight collagen hydrolysate in rats. J Agric Food Chem.

[CR519] Whittingham TS, Lipton P (1981). Cerebral synaptic transmission during anoxia is protected by creatine. J Neurochem.

[CR252] Willett W, Rockström J, Loken J (2019). Food in the anthropocene: the EAT-Lancet Commission on healthy diets from sustainable food systems. Lancet.

[CR520] Wilken B, Ramirez JM, Probst I (1998). Creatine protects the central respiratory network of mammals under anoxic conditions. Pediatr Res.

[CR253] Wright CE, Tallan HH, Lin YY (1986). Taurine: biological update. Annu Rev Biochem.

[CR254] Wu G (2009). Amino acids: metabolism, functions, and nutrition. Amino Acids.

[CR255] Wu G (2013). Amino acids: biochemistry and nutrition.

[CR256] Wu G (2016). Dietary protein intake and human health. Food Funct.

[CR257] Wu G (2018). Principles of animal nutrition.

[CR258] Wu G, Meininger CJ (2000). Arginine nutrition and cardiovascular function. J Nutr.

[CR259] Wu G, Morris SM (1998). Arginine metabolism: nitric oxide and beyond. Biochem J.

[CR260] Wu G, Meininger CJ, Knabe DA (2000). Arginine nutrition in development, health and disease. Curr Opin Clin Nutr Metab Care.

[CR261] Wu J, Fujioka M, Sugimoto K (2004). Assessment of effectiveness of oral administration of collagen peptide on bone metabolism in growing and mature rats. J Bone Miner Metab.

[CR262] Wu G, Bazer FW, Cudd TA (2007). Pharmacokinetics and safety of arginine supplementation in animals. J Nutr.

[CR263] Wu G, Bazer FW, Burghardt RC (2011). Proline and hydroxyproline metabolism: implications for animal and human nutrition. Amino Acids.

[CR264] Wu G, Wu ZL, Dai ZL (2013). Dietary requirements of "nutritionally nonessential amino acids" by animals and humans. Amino Acids.

[CR265] Wu G, Fanzo J, Miller DD (2014). Production and supply of high-quality food protein for human consumption: sustainability, challenges and innovations. Ann NY Acad Sci.

[CR266] Wu G, Cross HR, Gehring KB (2016). Composition of free and peptide-bound amino acids in beef chuck, loin, and round cuts. J Anim Sci.

[CR267] Wu ZL, Hou YQ, Dai ZL (2019). Metabolism, nutrition and redox signaling of hydroxyproline. Antioxid Redox Signal.

[CR268] Wu G, Bazer FW, Lamb GC, Bazer FW, Lamb GC, Wu G (2020). Significance, challenges and strategies of animal production. Animal agriculture: challenges, innovations, and sustainability.

[CR269] Wyss M, Kaddurah-Daouk R (2000). Creatine and creatinine metabolism. Physiol Rev.

[CR521] Wyss M, Schulze A (2002). Health implications of creatine: can oral creatine supplementation protect against neurological and atherosclerotic disease?. Neuroscience.

[CR522] Xu YJ, Arneja AS, Tappia PS (2008). The potential health benefits of taurine in cardiovascular disease. Exp Clin Cardiol.

[CR270] Xu S, He M, Zhong M (2015). The neuroprotective effects of taurine against nickel by reducing oxidative stress and maintaining mitochondrial function in cortical neurons. Neurosci Lett.

[CR271] Yan SL, Wu ST, Yin MC (2009). Protective effects from carnosine and histidine on acetaminophen-induced liver injury. J Food Sci.

[CR272] Yazaki M, Ito Y, Yamada M (2017). Oral ingestion of collagen hydrolysate leads to the transportation of highly concentrated Gly-Pro-Hyp and its hydrolyzed form of Pro-Hyp into the bloodstream and skin. J Agric Food Chem.

[CR273] Yeum K-J, Orioli M, Regazzoni L (2010). Profiling histidine dipeptides in plasma and urine after ingesting beef, chicken or chicken broth in humans. Amino Acids.

[CR274] Yu YM, Yang RD, Matthews DE (1985). Quantitative aspects of glycine and alanine nitrogen metabolism in postabsorptive young men. J Nutr.

[CR523] Zapara TA, Simonova OG, Zharkikh AA (2004). Seasonal differences and protection by creatine or arginine pretreatment in ischemia of mammalian and molluscan neurons in vitro. Brain Res.

[CR275] Zhang X, Song L, Cheng X (2011). Carnosine pretreatment protects against hypoxia-ischemia brain damage in the neonatal rat model. Eur J Pharmacol.

[CR276] Zhang Z, Zhao M, Wang J (2011). Oral administration of skin gelatin isolated from chum salmon (*Oncorhynchus keta*) enhances wound healing in diabetic rats. Mar Drugs.

[CR277] Zhang Z, Wang J, Ding Y (2011). Oral administration of marine collagen peptides from chum salmon skin enhances cutaneous wound healing and angiogenesis in rats. J Sci Food Agric.

[CR278] Zhou Y, Holmseth S, Guo C (2012). Deletion of the γ-aminobutyric acid transporter 2 (GAT2 and SLC6A13) gene in mice leads to changes in liver and brain taurine contents. J Biol Chem.

[CR279] Zhu S, Huang M, Feng G (2018). Gelatin versus its two major degradation products, prolyl-hydroxyproline and glycine, as supportive therapy in experimental colitis in mice. Food Sci Nutr.

